# Parvalbumin Neuron–Targeted Loss of Alzheimer’s Disease Risk Gene *BIN1* Is Insufficient to Drive Cognitive or Network Excitability Changes

**DOI:** 10.1523/ENEURO.0304-25.2026

**Published:** 2026-03-25

**Authors:** M. Natalie Davis, Mary Bullock, Aanishaa Jhaldiyal, Niya Holifield, Karim Mikhail, Yuliya Voskobiynyk, Rachael M. Vollmer, George C. Prendergast, Kelli Lauderdale, Jorge J. Palop, Karen L. Gamble, Erik D. Roberson

**Affiliations:** ^1^Department of Neurology, Heersink School of Medicine, The University of Alabama at Birmingham, Birmingham, Alabama 35294; ^2^Killion Center for Neurodegeneration and Experimental Therapeutics, The University of Alabama at Birmingham, Birmingham, Alabama 35294; ^3^Alzheimer’s Disease Center, The University of Alabama at Birmingham, Birmingham, Alabama 35294; ^4^Department of Psychiatry, Heersink School of Medicine, The University of Alabama at Birmingham, Birmingham, Alabama 35294; ^5^Lankenau Institute for Medical Research, Wynnewood, Pennsylvania 19096; ^6^Gladstone Institute of Neurological Disease, San Francisco, California 94158

**Keywords:** Alzheimer's disease, BIN1, network hyperexcitability, parvalbumin

## Abstract

*Bridging integrator 1* (*BIN1*) is one of the strongest genetic risk factors for Alzheimer’s disease (AD), yet its function in the brain and role in AD remain unclear. Neuronal *BIN1* isoform levels are decreased in AD, and recent data show an important role of *BIN1* in inhibitory neurons. Inhibitory neurons are key regulators of cognition and network excitability, with parvalbumin-expressing (PV) neurons as the most abundant subtype. We tested the hypothesis that loss of BIN1 from PV neurons contributes to AD-related cognitive dysfunction and network hyperexcitability. We generated a cell type–specific conditional knock-out mouse line, *Bin1-*pvKO, and examined mice of both sexes. These mice showed few behavioral differences when assessed with traditional or machine learning–based behavioral tests, with only a slight reduction in exploratory behavior in aged cohorts. *Bin1-*pvKO mice showed no significant differences in network excitability on measures of induced seizure susceptibility and spiking on cortical electroencephalographic recordings. Finally, *Bin1-*pvKO mice exhibited no major differences in power spectral analysis of cortical electroencephalographic recordings, with only a modest reduction in delta power at high activity levels. These findings suggest that BIN1 loss in PV neurons alone is insufficient to drive the cognitive and network dysfunction observed in AD models. While these results do not exclude a role of BIN1 in PV neurons in AD models, if combined with a “second hit” or alterations in other cell types, they indicate that BIN1 loss in PV neurons alone does not recapitulate key AD-related phenotypes.

## Significance Statement

Alzheimer's disease (AD) is the leading cause of dementia but lacks highly effective treatments. Genetic variants at the *bridging integrator 1* (*BIN1*) locus, present in ∼40% of the population, increase AD risk, but BIN1's role in the brain remains unclear. Understanding how BIN1 contributes to AD may yield new therapeutic targets. This study investigates the impact of BIN1 loss in parvalbumin-expressing neurons, an inhibitory neuron subtype vulnerable in AD. We show that BIN1 deletion in these cells does not reproduce cognitive or network abnormalities seen in AD models. These findings refine our understanding of BIN1's cell type–specific functions and highlight the need to explore its role in other types of inhibitory neurons or in combination with additional risk factors.

## Introduction

Alzheimer's disease (AD) is a complex neurodegenerative disorder of uncertain cause in most cases. However, genetics plays an important role in disease risk and progression, even in sporadic cases. *Bridging integrator 1* (*BIN1*) is a leading genetic risk factor for AD, with a population attributable risk second only to *APOE* (Seshadri, 2010; Lambert et al., 2013; Kunkle et al., 2019; Bellenguez et al., 2022). Given that ∼40% of the population carries *BIN1* risk alleles, it is important to understand the mechanisms by which BIN1 contributes to AD.

Several recent studies have pointed to a role of BIN1 at the synapse. BIN1 is present in both pre- and postsynaptic compartments, contributing to synaptic transmission in multiple ways (Daudin et al., 2018; De Rossi et al., 2020; Schürmann et al., 2020). Functionally, BIN1 affects neuronal activity, as shown by alterations in local field potential activity, excitatory and inhibitory postsynaptic currents, and presynaptic vesicular release (De Rossi et al., 2020; Schürmann et al., 2020; Voskobiynyk et al., 2020). BIN1 also directly interacts with various membrane receptors and channels, such as AMPA-type glutamate receptor subunits and L-type voltage-gated calcium channels, potentially regulating their synaptic expression and contributing to effects on neuronal activity (Schürmann et al., 2020; Voskobiynyk et al., 2020; Saha et al., 2024).

BIN1-dependent effects on neuronal activity and synapses could contribute to the network hyperexcitability seen in AD. Network hyperexcitability is a key feature of AD, seen in both patients and models of AD (Palop et al., 2007; Busche et al., 2008; Vossel et al., 2013; Šišková et al., 2014; Ghatak et al., 2019; Targa Dias Anastacio et al., 2022). AD patients show higher prevalence of epileptiform activity, which is associated with faster cognitive decline (Vossel et al., 2013; Vossel et al., 2016). Additionally, there appears to be a positive feedback loop, in which Aβ increases network hyperexcitability, and network hyperexcitability, in turn, also enhances Aβ deposition (Cirrito et al., 2005; Busche and Konnerth, 2016; Tombini et al., 2021; Tok et al., 2022).

Network hyperexcitability results from excitatory/inhibitory (E/I) imbalance, and inhibitory neuron dysfunction appears to be an important contributor (Palop et al., 2006; Busche and Konnerth, 2016; Maestú et al., 2021; Gabitto et al., 2024). GABA levels and GABA_A_ receptors are decreased in the brains of AD patients (Limon et al., 2012; Bai et al., 2015). Additionally, inhibitory neurons are selectively impaired in many models of AD and show susceptibility to Aβ toxicity (Krantic et al., 2012; Lei et al., 2016). Inhibitory neuron impairment leads to abnormal firing patterns, altered network excitability, and widespread circuit dysfunction (Palop et al., 2007; Verret et al., 2012; Hazra et al., 2013). Parvalbumin-expressing (PV) neurons are the most abundant type of inhibitory neuron and are vulnerable in AD (Arai et al., 1987; Satoh et al., 1991; Kumar et al., 2024). PV neurons help maintain E/I balance and control network oscillatory activity. Specifically, PV neurons control gamma oscillatory activity, which increases during memory encoding and decreases in AD (Hu et al., 2014; Hijazi et al., 2023). Dysfunction of PV neurons likely contributes to the overall inhibitory dysfunction seen in AD, but the extent of their contribution remains unknown.

BIN1 has many isoforms that are differentially expressed in various cell types (Prokic et al., 2014; Dourlen et al., 2025), which may explain why there has been conflicting data on how BIN1 expression changes in AD (Chapuis et al., 2013; Glennon et al., 2013; Holler et al., 2014; De Rossi et al., 2016). However, differential transcript expression across multiple patient cohorts shows decreased neuronal BIN1 isoforms in AD patients (Chapuis et al., 2013; Glennon et al., 2013; Holler et al., 2014; De Rossi et al., 2016; Marques-Coelho et al., 2021). Given that BIN1 is a synaptic protein that controls excitability and is reduced in neurons in AD and that AD is characterized by hyperexcitability potentially driven by PV neurons, we asked whether loss of BIN1 in PV neurons mimics the phenotypes observed in AD mouse models.

To answer this question, we generated a PV-Cre:*Bin1*^flox/flox^ (*Bin1*-pvKO) mouse model to examine the effects of loss of murine *Bin1* from PV neurons. Because of the role of PV neurons in cognitive function and network hyperexcitability, we focused on these phenotypes in our model. Overall, this study will provide insights into the cell type–specific effects of BIN1 and how these contribute to the network changes seen in AD.

## Materials and Methods

### Mice

*Bin1*^flox/flox^ mice (Chang et al., 2007) were crossed with PV-Cre mice (RRID:IMSR_JAX:017320; Hippenmeyer et al., 2005) for two generations to produce PV-Cre:*Bin1*^flox/flox^ (*Bin1-*pvKO) mice and sibling *Bin1*^flox/flox^ controls. These mice were on a congenic C57BL/6J background. Both male and female mice were used in the experiments, and siblings served as controls in each experiment. *Bin1*^flox/flox^ mice were also crossed with Nestin-Cre mice (RRID:IMSR_JAX:00377) for knock-out validation (Tronche et al., 1999). Additionally, a small group of hAPPJ20 mice (RRID:MMRRC_034836-JAX; Mucke et al., 2000) were used for EEG validation. All mice were group-housed in an Association for Assessment and Accreditation of Laboratory Animal Care International accredited facility on a 12 h light/dark cycle (zeitgeber time or ZT 0, lights on at 6:00 A.M. CST) with *ad libitum* access to food and water (NIH-31 Open Formula, LabDiet). All experiments were approved by the Institutional Animal Care and Use Committee of the University of Alabama at Birmingham.

Altogether, we tested a total of 286 mice across all experiments (152 controls, 134 *Bin1*-pvKO). For behavior and PTZ, we tested 24–35 mice per group, including both males and females, across four separate cohorts, two at 3–7 months old and two at 16–20 months old. For EEG, we implanted 13 mice per group, including both males and females; seven mice per group passed the exclusion criteria for analysis (detailed below in the EEG analysis). Weights were recorded for all analyzed mice prior to experimentation to include in body weight analysis. Subsets of these cohorts were used for model validation in fluorescent in situ hybridization, Western blots, and Nissl staining. All mice from the line were used for survival analysis.

### Single-molecule fluorescent in situ hybridization (smFISH)

smFISH was performed using BaseScope and RNAscope Multiplex Fluorescent V2 Assays [Advanced Cell Diagnostics (ACD)]. Flash-frozen brain hemispheres were sectioned at 20 µm on a Leica CM 1850 UV cryostat (RRID:SCR_025401), collected onto SuperFrost Plus slides (Thermo Fisher Scientific), and immediately refrozen at −80°C. For pretreatment, the tissue was removed from −80°C and immediately fixed with prechilled 10% neutral buffered formalin at 4°C, dehydrated with ethanol, and treated with hydrogen peroxide and protease III (ACD). Next, slides were incubated for 2 h at 40°C with a custom BaseScope probe (*BA-Mm-Bin1-2EJ,* catalog #709321) targeting exon 3 of *Bin1* (346–442 of NM_009668.2) to recognize the presence or absence of the floxed exon. Amplification of the probe was performed at 40°C, and development using BaseScope Fast RED A and B was performed at room temperature. Subsequently, RNAscope was performed by incubating the tissue in a mixture of RNAscope probes (*Mm-Pvalb* catalog #421931; *Mm-Gad1-C3*, catalog #400951-C3; ACD) for 2 h at 40°C, followed by fluorescent amplification. Slides were coverslipped with ProLong Gold Antifade Mountant with DAPI (Thermo Fisher Scientific), and images were obtained using an Olympus VS200 slide scanner (RRID:SCR_024783) or a Nikon A1R Confocal Laser Scanning Microscope (RRID:SCR_020317). A custom CellProfiler Image Analysis Software (RRID:SCR_007358) pipeline was used to identify regions of interest (ROIs) by cell markers (*Pvalb* and *Gad1*) and to quantify the percent area of *Bin1* signal within the ROIs (https://github.com/robersonlab-uab/Parvalbumin-BIN1-2026.git).

### Immunofluorescence

Immunofluorescence was performed on saline-perfused brains that were drop-fixed in 4% PFA, cryoprotected in 30% sucrose, and then sectioned at 30 µm on a sliding microtome (Leica Biosystems). These sections were washed in phosphate-buffered saline (PBS) to remove residual cryoprotectant and then permeabilized in PBST (PBS containing 0.1% Tween-20). Antigen retrieval was performed by incubating sections for 20 min in a low-pH citrate buffer, pH 6.0, heated to 95°C, followed by a 5 min incubation in 0.1% SDS to enhance antigen accessibility further. Sections were subsequently incubated for 60 min at room temperature in blocking buffer consisting of 0.5% fish gelatin, 10% goat serum, 3% bovine serum albumin, and 0.1% Triton X-100 prepared in 1× PBS and was filter-sterilized before use. Primary antibodies were diluted in blocking buffer and incubated overnight at 4°C. The following primary antibodies were used: BIN1 (Proteintech catalog #14647-1-AP, RRID:AB_2243396, 1:200), parvalbumin (Synaptic Systems catalog #195 308, RRID:AB_2927389, 1:1,000), and NeuN (Millipore catalog #ABN91, RRID:AB_11205760, 1:1,000). Alexa Fluor-conjugated secondary antibodies were applied (1:500) in 1× PBST and incubated for 1 h at room temperature, protected from light. Sections were subsequently counterstained with DAPI (Thermo Fisher Scientific catalog #PI62249, 1:1,000) to visualize nuclei. To reduce tissue autofluorescence, sections were treated with TrueVIEW Autofluorescence Quenching Solution (Vector Laboratories, SP-8400-15) for 2 min at room temperature. Finally, sections were mounted using ProLong Gold Glass Antifade Mountant (Thermo Fisher Scientific catalog #P36930) and allowed to cure prior to imaging. Confocal images were acquired using a Zeiss LSM 800 microscope equipped with a 63× objective, using a 1.1× digital zoom. Images were collected at a voxel size of 0.094 × 0.094 × 0.3 µm. Three-dimensional reconstruction and image analysis were performed using Imaris (Bitplane). Details of surface rendering can be found at https://doi.org/10.17504/protocols.io.dm6gp1oy8gzp/v1.

### Western blot

Cortices were dissected from flash-frozen brain hemispheres, then homogenized in Tris-buffered saline with protease inhibitor (1:100; Halt 78429). Protein concentrations were measured using a bicinchoninic acid (Pierce 23225) assay, and equal amounts of protein were loaded onto 4–12% Bis–Tris gels (Invitrogen WG1403A) for electrophoresis. Proteins were transferred onto PVDF–FL membrane (Millipore IPFL00010), blocked with Odyssey blocking buffer (LI-COR, 927–40,000), and immunoblotted with anti-BIN1 99D (1:1,000, Millipore catalog # 05-449, RRID:AB_309738) and anti-GAPDH (1:5,000, Millipore catalog #MAB374, RRID:AB_2107445) primary antibodies and Alexa Fluor 700- or 800-conjugated goat secondary antibodies (1:20,000, LI-COR). Blots were visualized on a LI-COR Odyssey Classic Imager (RRID:SCR_023765) and quantified using LI-COR Image Studio Lite (v. 5.2.5, RRID:SCR_013715).

### Behavioral tests

Four cohorts of mice, two at each age (3–7 and 16–20 months), were analyzed for behavior. All mice were habituated to the rooms where the behavioral tests were performed for 1 h before testing in dim light. Genotypes were randomly distributed in a behavioral order, and investigators remained blind to genotypes throughout behavioral testing. Mice were excluded from analysis if they did not complete the task as outlined for each assay.

#### Elevated plus maze

The elevated plus maze consists of two open arms (with no walls) and two closed arms (enclosed by walls on three sides). During testing, the mouse was placed in the center of the maze and allowed to explore for 5 min. Motion was tracked using either the MED Associates Activity Monitor software (RRID:SCR_014296) or EthoVision XT (RRID:SCR_000441) video tracking software, and data were analyzed for total distance traveled, time spent in each arm, the number of explorations into each arm, and the number of entrances into each arm.

#### Open-field maze

The open-field maze involves allowing the mouse to explore a 40 × 40 cm box for 10 min and recording motion using the MED Associates Activity Monitor software (RRID:SCR_014296) or EthoVision XT (RRID:SCR_000441) video tracking software. Data were analyzed for total distance traveled, distance by time, and time spent in the center versus the outer areas.

#### Y maze

The Y maze consists of three 15-inch-long, 3.5-inch-wide, and 5-inch-high arms. Mice were placed in the center hub of the maze and allowed to explore freely for 5 min with video recording. The EthoVision XT (RRID:SCR_000441) software was used to track total distance traveled, arm entrances and explorations, and alternations. Mice were excluded from analysis if they failed to perform a successful alternation (*n* = 1 mouse excluded).

#### Contextual fear conditioning

Med Associates Video Fear Conditioning boxes were used for the contextual fear conditioning paradigm, which consisted of 1 d of training and 1 d of testing 24 h later. During the training phase, mice were placed inside the chambers and allowed to explore for 3 min (baseline), before receiving three auditory cues (20 s duration; white noise, 75 dB) followed by mild footshocks (0.5 mA, 2 s duration) 1 min apart, with 2 min of association time after the last footshock. For testing, each mouse was returned to the same chamber 24 h later and video recorded for 5 min. The percentage of time freezing was calculated (motion threshold, 20; minimum freeze duration, 1 s; method, linear) using the manufacturer's software Med Associates Video Freeze Software (RRID:SCR_014574).

#### Pose estimation

Mice were placed in a circular open field (16 inch diameter) with a clear bottom and video recorded from below using a FLIR Backfly PoE GigE camera at 30 frames per second (fps) for 1 h. Twenty frames per recording were labeled for the following key points: nose, all four paws, belly, and the base of the tail. These labeled images were used to train a DeepLabCut (DLC; RRID:SCR_021391) network for one million iterations (Mathis et al., 2018; Nath et al., 2019). Pose trajectories were derived from egocentric coordinates obtained through DLC pose estimation. For egocentric alignment, the anterior reference point was determined using the nose, while the posterior reference point was identified as the base of the tail. A principal component analysis (PCA) of these egocentrically aligned key point time series was extracted from DLC and used to create a model in Keypoint-MoSeq (KPMS; RRID:SCR_025032; Weinreb et al., 2024).

#### Behavioral clustering

Adhering to the established procedures outlined in the KPMS repository (https://github.com/dattalab/keypoint-moseq/), we executed the standard workflow to predict syllables for each video frame. A hyperparameter, kappa, was configured at a value of 10 million. Subsequently, an autoregressive hidden Markov model was fit to the pose trajectory data over 50 iterations. Finally, a complete KPMS model was created using the provided documentation and trained for 500 iterations. KPMS outputs of frame-by-frame syllable usage were used to create a frequency distribution for each mouse, and the similarity dendrogram was used to compare the clustering of these identified syllables. Distance traveled was tracked using the ezTrack (RRID:SCR_021496) Location Tracking Module (Pennington et al., 2019).

### Pentylenetetrazole-induced seizures

Pentylenetetrazole (PTZ; Sigma-Aldrich) was dissolved in sterile saline at a concentration of 4 mg/ml for younger cohorts (3–7 months) and 3 mg/ml for older cohorts (16–20 months). PTZ was administered at a dose of 40 mg/kg (for younger mice) or 30 mg/kg (for older mice) of body weight, injected intraperitoneally, with genotypes randomly distributed in and injection order. The injected mice were placed in a cage for 20 min of observation by an investigator who was blinded to the genotype. The following scale of seizure stages was used: 0, normal behavior; 1, immobility; 2, generalized spasm, tremble, or twitch; 3, tail extension; 4, forelimb clonus; 5, generalized clonic activity; 6, bouncing or running seizures; 7, full tonic extension; and 8, death (Racine, 1972; Löscher et al., 1991). The latency to reach each stage and the maximum stage reached were recorded for each mouse.

### Electrocorticography recordings

Note: the recordings performed here were cortical, making them electrocorticographic recordings; however, the more commonly used terminology of EEG is used throughout the paper for simplicity.

#### Surgeries

Mice were anesthetized with 5% isoflurane and placed in a stereotaxic frame, where they were maintained on 2.5% isoflurane during device implantation. Two burr holes were drilled into the skull, one 2 mm posterior to the bregma and 1.3 mm lateral to the midline for recording above the parietal cortex, with the other 6 mm posterior to the bregma and 1 mm lateral to the midline for the reference electrode over the cerebellar cortex. Screws attached to the EEG leads of the wireless telemetry device (HD-X02, Data Sciences International) were screwed into the burr holes and secured with dental cement (Flow-It ALC, Pearson Dental). Two electromyography (EMG) electrodes were placed in the deep parasagittal cervical muscles. The biocompatible transmitter was placed subcutaneously over the left flank per manufacturer’s recommendations. Once all parts of the device were positioned, the skin was closed over the entire apparatus. For analgesia, buprenorphine (0.1 mg/kg) and carprofen (5 mg/kg) were administered, along with topical lidocaine ointment (5%) at the incision site. Mice were allowed to recover for at least 7 d before recordings.

#### Recordings

Single-housed mice in their home cages were placed in light-controlled boxes on top of radio-frequency receivers that transmitted signals to an acquisition computer over Ethernet (Data Sciences International). Mice were allowed to habituate to their new environment for at least 48 h before recording. Mice were maintained on a standard light/dark cycle (6 A.M./6 P.M.). Simultaneous EEG, EMG, body temperature, and locomotor activity signals were continuously recorded for 7 d using the Ponemah (RRID:SCR_017107) software, sampled at 500 Hz.

#### Exclusion criteria

Mice were excluded from analysis if (1) there were issues that prevented obtaining usable data (i.e., death, poor recovery from surgery, damaged devices, excessive noise outside of physiological range) or (2) there was movement-induced noise that made recordings uninterpretable, defined by an increase in delta power with increasing locomotor activity, as delta activity should decrease with activity in awake and moving animals. For this study, 26 mice (13 per group) were implanted with EEG devices, and 6 per group were excluded by these criteria, leaving 7 per group in the final analysis.

#### Spike analysis

The Spike2 Software (RRID:SCR_000903, v10.21) was used to analyze spiking activity. First, a high-pass FIR digital filter was applied to EEG recordings to reduce low-frequency noise below 6.3 Hz. Spikes were automatically detected as any signal ≥10 times the standard deviation of baseline EEG and then hand-scored to exclude noise and include only waveforms in the range of 15–70 ms.

#### Spectral analysis

Data were processed using a custom MATLAB script as outlined in the attached protocol (https://doi.org/10.17504/protocols.io.bp2l6dzbzvqe/v1). Briefly, raw data were cleaned by removing large artifacts (−2 to +2 mV cutoff). A high-pass filter of 0.5 Hz was applied to the EEG data before running a fast Fourier transformation with a 500 ms window to calculate the median oscillatory power for the spectrogram (0.5–80 Hz) in 10 s bins. These bins were averaged every minute for the entire recording. The average raw power and average activity per minute were graphed per mouse, and a simple linear regression was run for each frequency band. These linear regressions were used to determine the relationship between activity and each power band by group. For time-based power analysis, the minute-bin averages were normalized to the tenth percentile of the total power of each mouse. Hour averages of the cleaned and normalized data were then calculated and used for 24 h cycle analysis. The percentage of total power was calculated and averaged across the entire recording for each mouse to compare the proportion of total power.

#### Sleep analysis

The first 24 h cycle of EEG recordings was scored in 10 s epochs as wake, NREM (nonrapid eye movement), or REM (rapid eye movement) using Sirenia Sleep Pro (RRID:SCR_022918, v.1.8.4). Epochs were scored based on wave activity (i.e., slow-/fast-wave activity) and depolarization size within the EEG, as well as the presence or absence of a signal in the activity and EMG channels. Wake was defined by a signal within the activity channel, small and random depolarizations within the EEG channel, and a distinct EMG signal. NREM was defined by the absence of activity, the presence of large depolarizations and slow-wave activity within the EEG, and minimal to no signal within the EMG. REM was defined by the absence of signal in the activity channel, the presence of medium-wave-like depolarizations within the EEG, and minimal EMG signal. Epochs containing more than one sleep stage criterion were scored as the stage that occupied most of the epoch. Artifacts (i.e., high amplitude depolarizations, loss of EEG signal, etc.) within NREM and REM were scored as such, but only significantly large artifacts in wake were scored as artifacts. Recordings with ≥5% artifacts were excluded from analysis. Sleep scores and EEG power for each stage were exported from Sirenia Sleep Pro software and then further analyzed using custom MATLAB scripts (https://doi.org/10.17504/protocols.io.bp2l6dzbzvqe/v1).

### Data analysis and statistics

All data were evaluated for normality and analyzed using parametric or nonparametric tests accordingly. Two-way ANOVAs assessed main effects and interactions. Where specified, linear mixed-effect models were used and fit by maximum likelihood with genotype as a fixed effect and controlling for mouse as a random effect: Mean ∼ Genotype + (1|Mouse). This model was run in RStudio (RRID:SCR_000432, v. 4.4.0) using the lme4 package. All other statistical tests were performed using GraphPad Prism 10 (RRID:SCR_002798, v. 10.4.1). All relevant statistical information can be found in Extended Data 1.

### Code accessibility

The code described in the paper is freely available online (https://doi.org/10.17504/protocols.io.bp2l6dzbzvqe/v1).

10.1523/ENEURO.0304-25.2026.d1 Data 1 **Statistics table.** Organized by Figure panel, showing description, test, n, relevant statistics, p-values, means, and standard errors. Download Data 1, DOCX file.

## Results

### Selective loss of *Bin1* from PV cells in *Bin1-*pvKO mice

**Figure 1.**
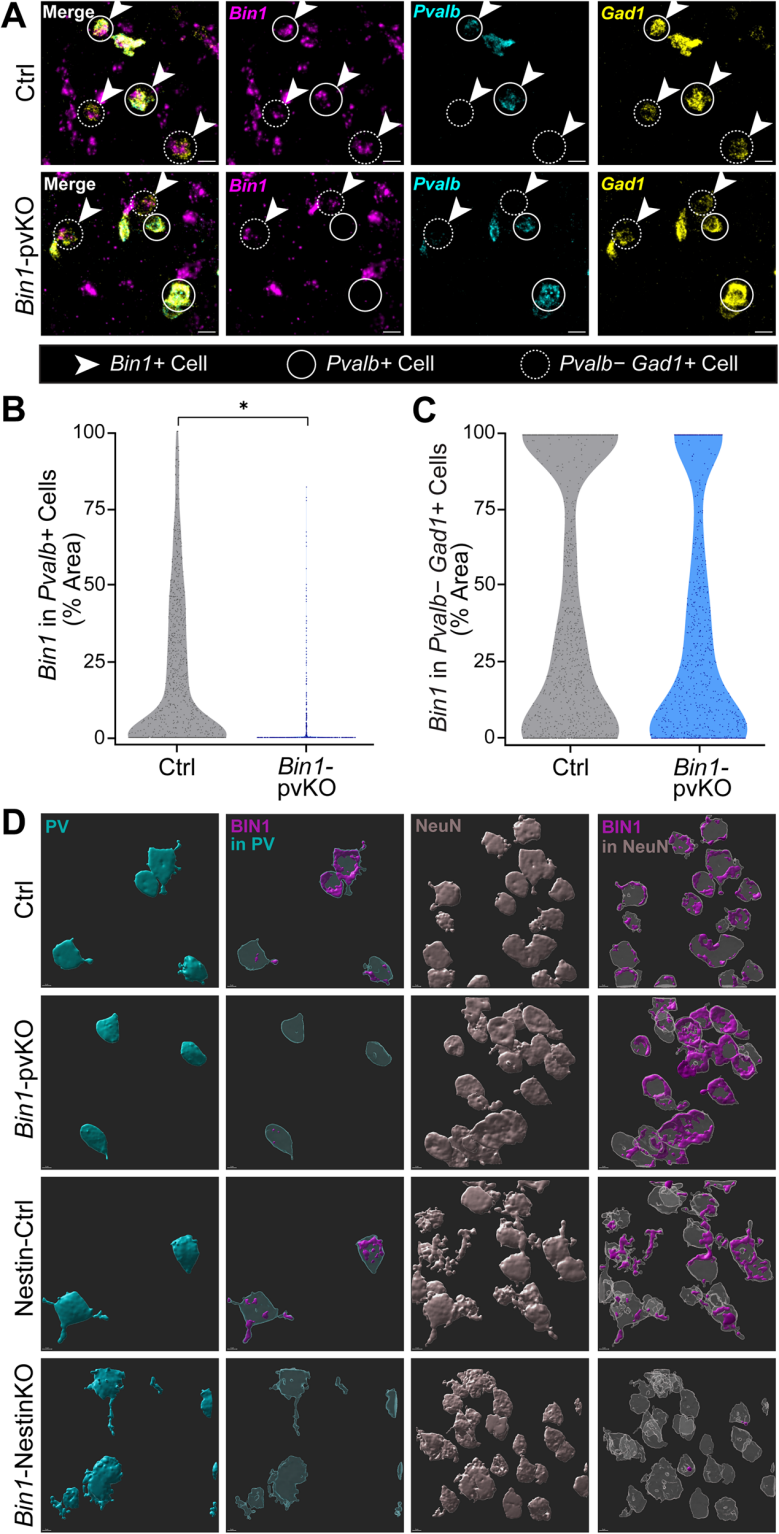
Selective loss of *Bin1* from PV cells in *Bin1*-pvKO mice. ***A***, High power images (60×) of smFISH in the cortex of 5-month-old mice, showing *Pvalb^+^* cells (cyan, representative cells in solid circles) and *Pvalb^−^ Gad1^+^* cells (yellow, representative cells in dotted circles) with *Bin1* (magenta, in representative cells with arrowheads). Whole-brain images of smFISH can be found in Extended Data Figure 1-2*A*, with regional zooms in Extended Data Figure 1-2*B*. ***B***, The percent area of *Pvalb^+^* cells occupied by *Bin1* signal in the cortex [analyzed by linear mixed-effect model with genotype as a fixed effect and controlling for mouse as a random effect; *β* *=* −1.42; SE *=* 0.26; *t*_(3)_ = −5.47; *p* = 0.012; *N* = 2–3 animals per group (523–1,149 cells per group), aged 5–7 months]. ***C***, The average percent area of *Pvalb^−^ Gad1^+^* cells occupied by *Bin1* signal in the cortex [analyzed by linear mixed-effects model with genotype as a fixed effect and controlling for mouse as a random effect; *β* *=* −0.23; SE *=* 0.33; *t*_(3)_ = −0.699; *p* = 0.5346; *N* = 2–3 animals per group (927–1,617 cells per group), aged 5–7 months]. ***D***, 3D rendering of cell type–specific visualization of Bin1 signal by immunohistochemistry in the cortex, showing PV ROIs (cyan), Bin1 signal in PV ROIs (magenta), NeuN ROIs (gray), and Bin1 signal in NeuN ROIs (magenta) in Ctrl, *Bin1-*pvKO, Nestin-Ctrl, and *Bin1-*Nestin-KO mice (aged 5–7 months). IHC without 3D renderings can be seen in Extended Data Figure 1-1. Total Bin1 protein levels measured by Western blotting shown in Extended Data Figure 1-2*C*,*D*.

To investigate the role of *Bin1* in PV neurons, we crossed homozygous *Bin1-*floxed mice with PV-Cre mice to selectively delete *Bin1* from PV neurons (*Bin1*-pvKO; Hippenmeyer et al., 2005; Chang et al., 2007). To confirm effective and selective knock-out, we performed smFISH using probes targeting the deleted *Bin1* exon (exon 3, which is present in all splice isoforms) along with cell type–specific markers for PV and Gad neurons. *Bin1* mRNA was selectively lost in PV cells, but not in other Gad cells (Fig. 1*A*). In *Bin1-*pvKO mice, average *Bin1* mRNA expression was reduced by ∼75% in PV cells, with a median expression level of zero (Fig. 1*B*), meaning most PV neurons lacked any detectable *Bin1* expression. There was no change in *Bin1* expression in other Gad cells, showing specific reduction in PV cells (Fig. 1*C*).

10.1523/ENEURO.0304-25.2026.f1-1 Figure 1-1 **Bin1 immunohistochemistry in *Bin1*-pvKO mice.** Representative immunofluorescence images from the cortex of Ctrl, *Bin1-*pvKO, Nestin-Ctrl, and *Bin1-*NestinKO mice showing DAPI (blue), NeuN (gray), PV (cyan), and Bin1 (magenta) signals individually and merged (aged 5–7 months). These are maximum intensity projections from the z-stack images used to generate the 3D reconstructions in Fig. 1D. Download Figure 1-1, TIF file.

**Figure 2.**
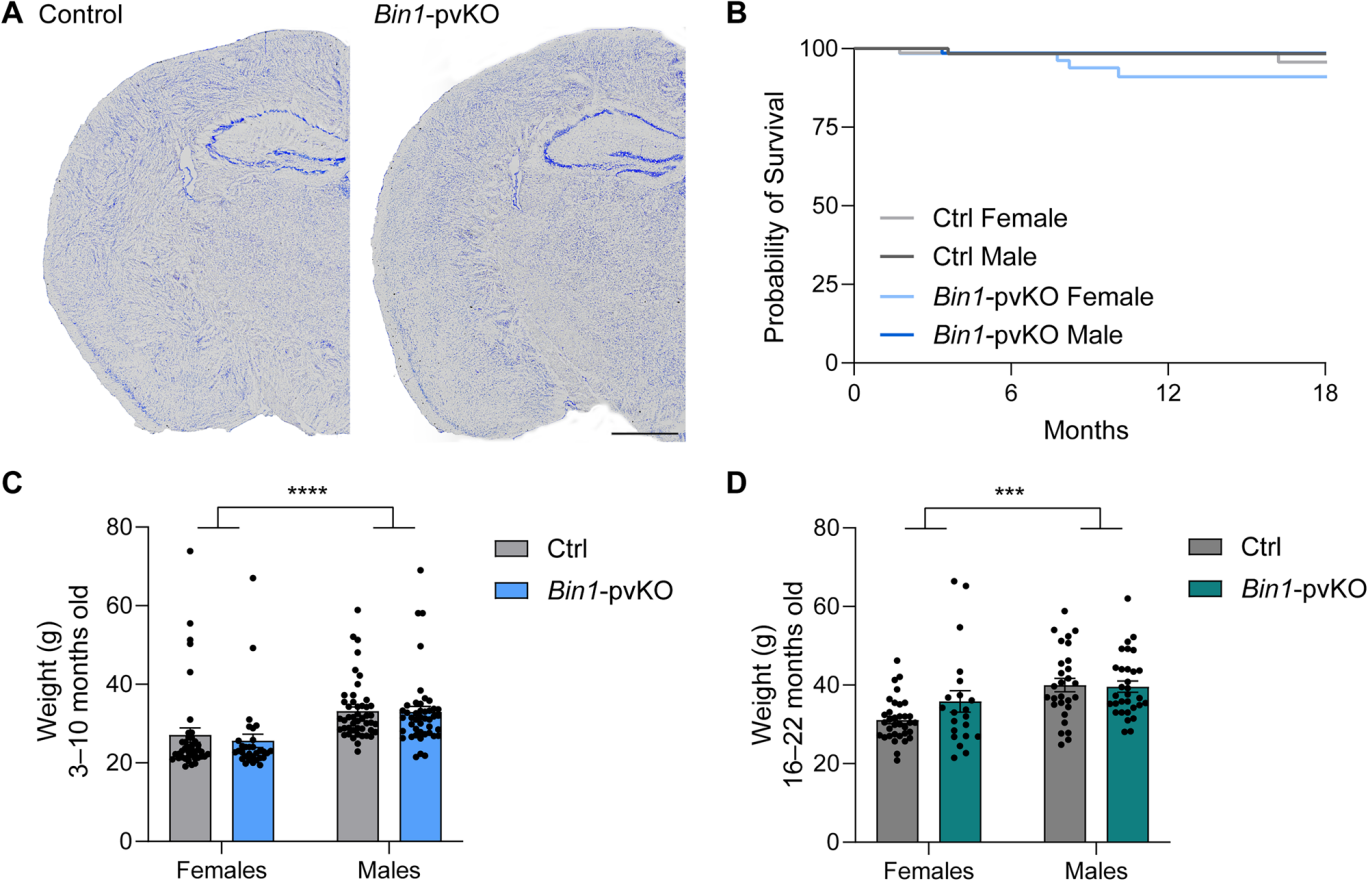
Loss of *Bin1* from PV neurons does not alter brain structure, survival, or body weight. ***A***, Nissl stain of Ctrl and *Bin1-*pvKO mice. ***B***, Survival curves of Ctrl and *Bin1-*pvKO mice split by sex (Mantel–Cox test, *χ*^2^ = 1.330; *p* = 0.7221; df = 3; *n* = 63–71 mice per group). ***C***, Body weight measurements of Ctrl and *Bin1-*pvKO mice across younger cohorts (3–10 months old), split by sex (two-way ANOVA; interaction, *F*_(1,158)_ = 0.1807; *p* = 0.6713; sex, *F*_(1,158)_ = 19.83; *p* < 0.0001; genotype, *F*_(1,158)_ = 0.2965; *p* = 0.5868). ***D***, Body weight measurements of Ctrl and *Bin1-*pvKO mice across aged cohorts (16–22 months old), split by sex (two-way ANOVA; interaction, *F*_(1,110)_ = 2.447; *p* = 0.1206; sex, *F*_(1,110)_ = 14.86; *p* *=* 0.0002; genotype, *F*_(1,110)_ = 1.745; *p* = 0.1892; Fisher’s LSD, females, *p* = 0.0503; males, *p* = 0.8593; Ctrl, *p* < 0.0001; *Bin1*-pvKO, *p* = 0.1256).

10.1523/ENEURO.0304-25.2026.f1-2 Figure 1-2 **Selective loss of *Bin1* from PV cells in *Bin1*-pvKO mice. A)** Low power images showing smFISH of *Bin1* (magenta) and *Pvalb* (cyan) of a control (top) and *Bin1-*pvKO (bottom) hemibrain, boxed insets for zoom in panel B. **B)** Higher power view of two PV neuron–dense regions (medial prefrontal cortex [mPFC] and reticular nucleus of the thalamus [nRT], boxed in panel A), showing total *Bin1* in the region, a mask created by *Pvalb* *+* cells, and the *Bin1* signal present only in the *Pvalb* mask, with control tissue on top and *Bin1-*pvKO tissue on bottom as in panel A. **C)** Full Western blot of Control (PV-Cre–) and *Bin1-*pvKO (PV-Cre+) cortical homogenate, probed for Bin1 and Gapdh. **D)** Quantification of total Bin1 signal normalized to Gapdh for each sample then normalized to Ctrl (unpaired t test, t (12) = 2.302, *p* = 0.0400, n = 7 per group, aged 5–7 months). Download Figure 1-2, TIF file.

We further validated this knock-out by examining Bin1 protein levels in PV and non-PV cells. Bin1 antibodies widely stain the neuropil, making cell type–specific detection difficult (De Rossi et al., 2016; Sudwarts et al., 2022; Extended Data Fig. 1-1). Therefore, we examined Bin1 signal in cell bodies by creating three-dimensional ROIs based on PV and NeuN staining. Bin1 signal was depleted from PV cells in *Bin1*-pvKO mice but remained in other NeuN^+^ cells (Fig. 1*D*). As a positive control, we crossed *Bin1*-floxed mice with Nestin-Cre mice to delete *Bin1* from all neurons. No Bin1 staining was observed in *Bin1*-Nestin-KO mice, confirming antibody specificity (Fig. 1*D*).

Because PV neurons make up a relatively small proportion of cells in the brain, *Bin1*-pvKO mice should have no major changes in overall Bin1 expression. As expected, there were no gross differences in *Bin1* mRNA levels at low magnification on smFISH (Extended Data Fig. 1-2*A*). However, looking closely at PV-dense regions, such as the medial prefrontal cortex and the reticular nucleus of the thalamus, the majority of *Pvalb^+^* cells in *Bin1-*pvKO mice lacked *Bin1* mRNA expression (Extended Data Fig. 1-2*B*). Additionally, total Bin1 protein levels were reduced by ∼15% in the cortex in *Bin1-*pvKO mice, consistent with selective reduction of Bin1 in the PV population (Extended Data Fig. 1-2*C*,*D*). Overall, these data show a significant and selective reduction of Bin1 from PV neurons at both the mRNA and protein levels.

### Selective loss of *Bin1* from PV neurons causes no overt health problems

Complete loss of *Bin1* is perinatally lethal due to cardiac toxicity (Muller et al., 2003), so we first determined whether cell type–specific reduction had any major adverse effects. Loss of *Bin1* from PV neurons alone did not alter overall brain architecture as assessed by Nissl staining (Fig. 2*A*). *Bin1-*pvKO mice also showed no difference in survival in either sex (Fig. 2*B*). There was an expected sex effect on body weight in both young and aged mice, with males having a higher average body weight than females (Fig. 2*C*,*D*). However, there was no genotype effect, except for a trend toward higher weight in older female *Bin1-*pvKO mice (Fig. 2*D*).

### Loss of *Bin1* from PV neurons decreases exploratory behavior with aging

**Figure 3.**
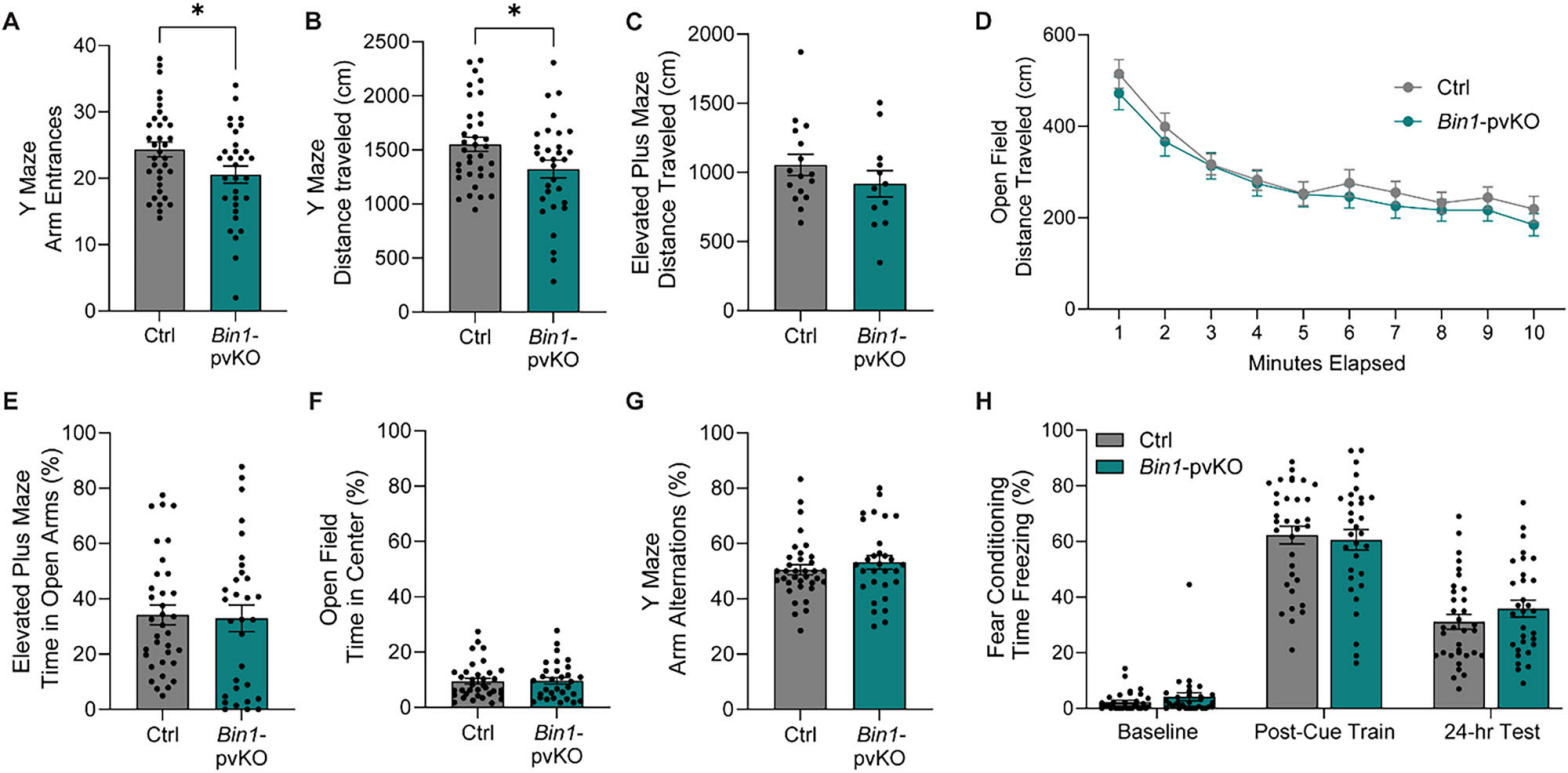
Older *Bin1*-pvKO mice show mildly decreased exploratory behavior. Behavioral screening of 16–20-month-old *Bin1-*pvKO mice showed a significant decrease in ***A***, the number of arm entrances in the Y maze (unpaired *t* test, *t*_(64)_ = 2.247; *p* = 0.0281; *n* = 31–35 per group; split by sex in Extended Data Figure 3-1*A*,*B*) distance traveled in the Y maze (unpaired *t* test, *t*_(64)_ = 2.220; *p* = 0.0300; *n* = 31–35 per group; split by sex in Extended Data Fig. 3-1*B*), but not in ***C***, distance traveled in the elevated plus maze (unpaired *t* test, *t*_(26)_ = 1.130; *p* = 0.2688; *n* = 12–16 per group), or ***D***, total distance traveled in the open field (two-way RM ANOVA; interaction effect, *F*_(9,576)_ = 0.3908; *p* = 0.9397; genotype effect, *F*_(1,64)_ = 0.5156; *p* = 0.4753; time effect, *F*_(1.808,115.7)_ = 61.76; *p* < 0.0001; *n* = 31–35 per group). *Bin1-*pvKO mice do not show differences in ***E***, the percentage of time spent in the open arms of the elevated plus maze (Mann–Whitney test, *U* = 506; *p* = 0.6435; *n* = 31–35 per group); ***F***, amount of time spent in the center of the open-field maze (Mann–Whitney test, *U* = 541; *p* = 0.9872; *n* = 31–35 per group); ***G***, spontaneous alternations in the Y maze (Mann–Whitney test, *U* = 452.5; *p* = 0.3432; *n* = 30–35 per group); or ***H***, the percentage of time freezing in contextual fear conditioning (two-way RM ANOVA; interaction effect, *F*_(2,126)_ = 0.8953; *p* = 0.4111; genotype effect, *F*_(1,63)_ = 0.4092; *p* = 0.5247; time effect, *F*_(1.736,109.3)_ = 298; *p* < 0.0001; *n* = 31–34 per group).

To assess how loss of *Bin1* from PV neurons affects behavior, we performed a battery of behavioral assays, starting with mice aged 16–20 months. Exploratory behavior was reduced in *Bin1*-pvKO mice in the Y maze, as seen by a decrease in the number of arm entrances (Fig. 3*A*) and total distance traveled (Fig. 3*B*). These effects were not driven by one sex, with similar trends in both males and females (Extended Data Fig. 3-1). However, there was no significant difference in the distance traveled in the elevated plus maze (Fig. 3*C*) or in the open field (Fig. 3*D*). This phenotype was not driven by differences in anxiety-related behaviors, as evidenced by the time spent in the open arms of the elevated plus maze (Fig. 3*E*) or in the center of the open field (Fig. 3*F*). Additionally, there were no differences in learning and memory, tested through spontaneous arm alternations in Y maze (Fig. 3*G*) and time freezing in contextual fear conditioning (Fig. 3*H*).

10.1523/ENEURO.0304-25.2026.f3-1 Figure 3-1 **Exploratory behavior in the Y Maze split by sex.** The differences seen in Y maze A) number of arm entrances (two-way ANOVA, interaction: F (1, 62) = 0.05641, *p* *=* 0.8131; genotype: F (1, 62) = 1.848, *p* *=* 0.1789; sex: F (1, 62) = 12.51, *p* *=* 0.0008) and B) total distance traveled (two-way ANOVA, interaction: F (1, 62) = 0.0002152, *p* *=* 0.9883; genotype: F (1, 62) = 1.923, *p* *=* 0.1705; sex: F (1, 62) = 9.577, *p* *=* 0.0030) were not driven by one sex. Download Figure 3-1, TIF file.

**Figure 4.**
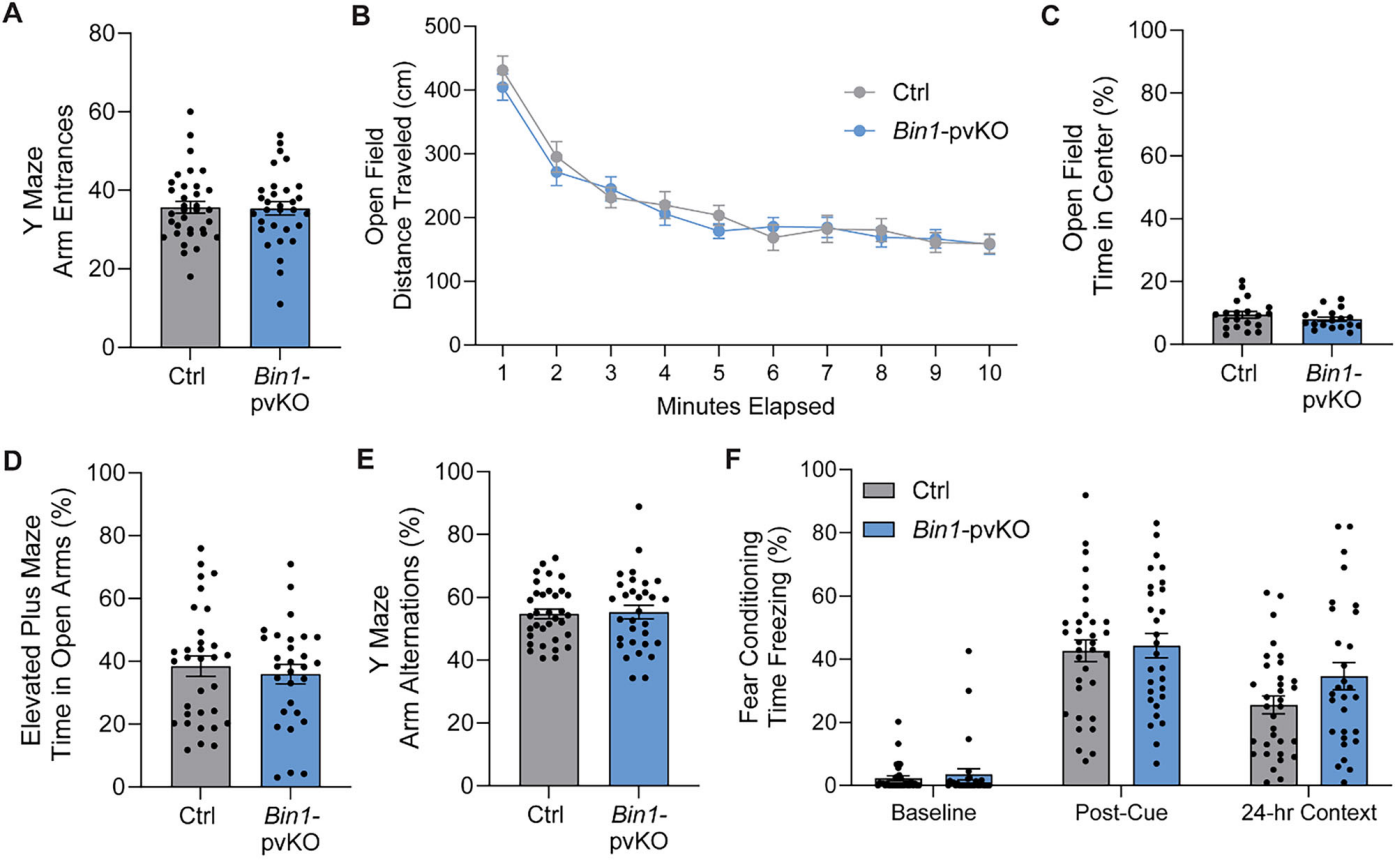
Young *Bin1*-pvKO mice do not exhibit behavioral alterations. *Bin1-*pvKO mice at 3–7 months showed no difference in ***A***, the number of arm entrances in the Y maze (unpaired *t* test, *t*_(63)_ = 0.1412; *p* = 0.8882; *n* = 31–34 per group); ***B***, total distance traveled in the open-field maze (two-way RM ANOVA; interaction effect, *F*_(9,558)_ = 0.5879; *p* = 0.8075; genotype effect, *F*_(1,62)_ = 0.1393; *p* = 0.7103; time effect, *F*_(3.383,209.7)_ = 56.91; *p* < 0.0001; *n* = 30–34 per group); ***C***, time spent in the center of the open-field maze (unpaired *t* test, *t*_(36)_ = 1.098; *p* = 0.2794; *n* = 18–20 per group); ***D***, the percentage of time spent in the open arms of the elevated plus maze (unpaired *t* test, *t*_(59)_ = 0.5556; *p* = 0.5806; *n* = 29–32 per group); ***E***, spontaneous alternations in the Y maze (unpaired *t* test, *t*_(63)_ = 0.1895; *p* = 0.8503; *n* = 31–34 per group); or ***F***, the percentage of time freezing in training or contextual testing of fear conditioning (two-way RM ANOVA, interaction effect, *F*_(2,120)_ = 1.651; *p* = 0.1961; genotype effect, *F*_(1,60)_ = 1.484; *p* = 0.2280; time effect, *F*_(1.985,119.1)_ = 143.2; *p* < 0.0001; *n* = 29–33 per group).

To determine if the decrease in exploratory behavior was a developmental phenotype, we tested younger mice with the same behavioral battery. At 3–7 months old, there were no differences in the number of arm entrances in the Y maze or the total distance traveled in the open field (Fig. 4*A*,*B*). Additionally, there was no change in anxiety-related behaviors, as seen in the percentage of time spent in the center of the open field (Fig. 4*C*) or in the open arms of the elevated plus maze (Fig. 4*D*). Finally, there was no change in learning and memory at this age, as seen by the percentage of spontaneous alternations in the Y maze (Fig. 4*E*) and time freezing in contextual fear conditioning (Fig. 4*F*). Overall, in these traditional assays, the behavior of *Bin1-*pvKO mice was unchanged from controls at 3–7 months old.

**Figure 5.**
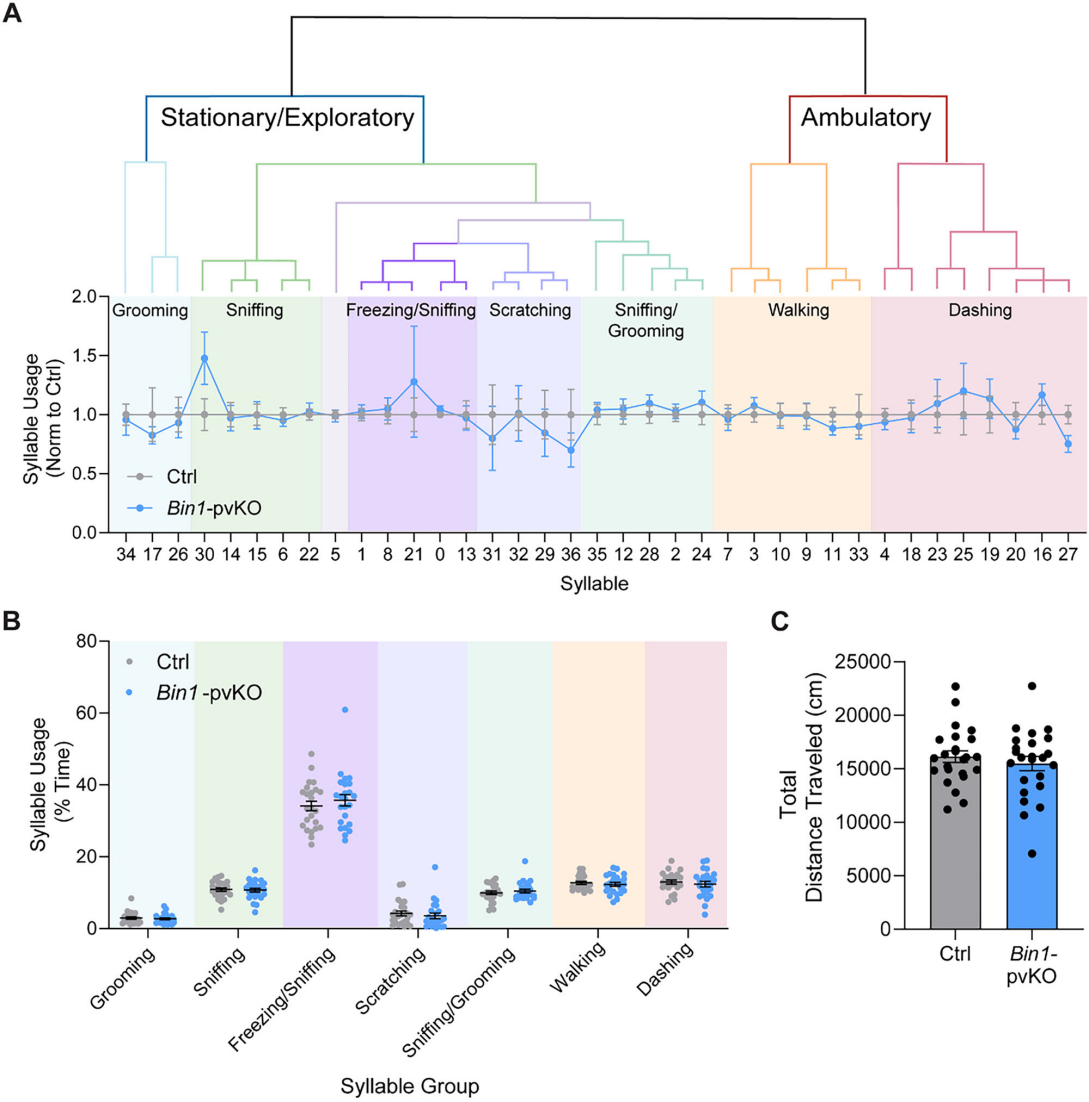
Machine learning–based pose estimation and behavioral clustering show no differences in *Bin1-*pvKO mice. ***A***, Similarity dendrogram from KPMS behavioral clustering analysis following DLC pose estimation trained network. Syllables were sorted by dendrogram clustering, grouped by behavior class, and normalized to control syllable usage (two-way RM ANOVA of syllable usage across genotypes; interaction effect, *F*_(36,1656)_ = 0.5730; *p* = 0.9806; effect of genotype, *F*_(1,46)_ = 0.004145; *p* = 0.9489; effect of syllable, *F*_(5.837,268.5)_ = 0.5730; *p* *=* 0.7471; *n* = 24 per group). ***B***, Sum of percent time spent in each syllable group, color coded and clustered from panel ***A*** (two-way RM ANOVA of syllable group usage; interaction effect, *F*_(6,276)_ = 0.4985; *p* = 0.8093; genotype effect, *F*_(1,46)_ = 0.02772; *p* *=* 0.8685; syllable effect, *F*_(1.960,90.16)_ = 349.1; *p* < 0.0001; *n* = 24 per group). ***C***, Total distance traveled in the hour-long recording session, as tracked by the ezTrack software (unpaired *t* test, *t*_(46)_ = 0.7510; *p* = 0.4565; *n* = 24 per group).

New computer-vision and machine-learning approaches provide a more sensitive and unbiased tool for behavioral analysis (Nath et al., 2019; Miller et al., 2024; Weinreb et al., 2024). To more sensitively characterize any behavioral differences in the 3–7-month-old *Bin1-*pvKO mice, we used machine learning–based pose estimation and behavior clustering software to identify and track short kinematic motifs, referred to as “syllables,” during spontaneous, undirected behavior. There were no differences in syllable usage in *Bin1-*pvKO mice (Fig. 5*A*), including when collapsed by PCA into a similarity dendrogram that was subsequently human-labeled to describe the types of behavioral syllables (Fig. 5*B*). Additionally, there were no differences in the total distance traveled across the entire recording (Fig. 5*C*). Overall, we conclude that *Bin1-*pvKO mice have no discernable behavioral abnormalities at young ages, even with sensitive machine learning algorithms, and develop a mild decrease in exploratory behavior with aging.

### Loss of *Bin1* from PV neurons does not significantly alter network hyperexcitability

**Figure 6.**
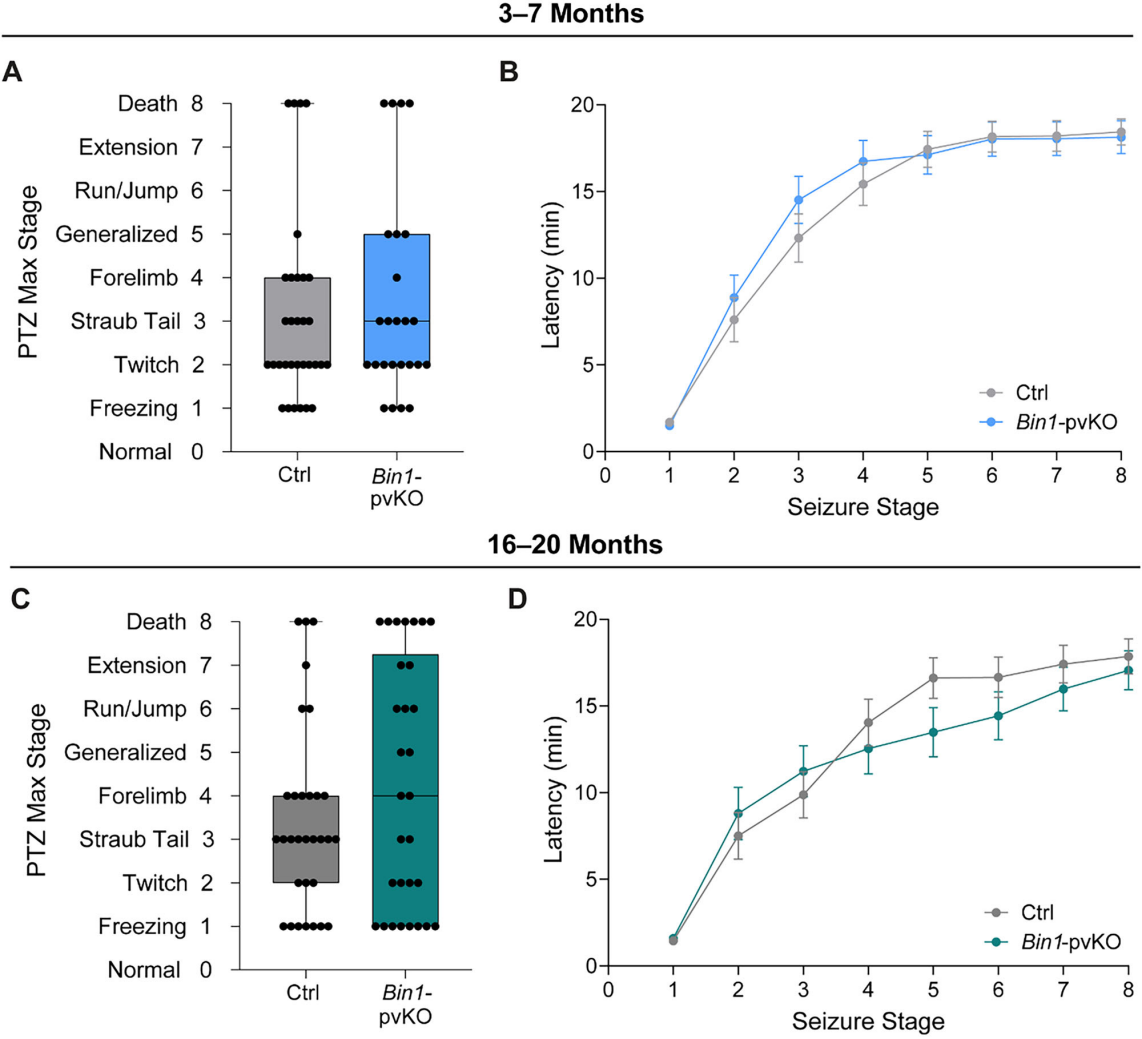
*Bin1-*pvKO mice do not show increased PTZ-induced seizure susceptibility. ***A***, Maximum seizure stage reached after PTZ (40 mg/kg) at 3–7 months (Mann–Whitney test, *U* = 371.5; *p* = 0.6426; *n* = 25–32 per group) and ***B***, latency to each stage (two-way RM ANOVA; interaction effect, *F*_(7,385)_ = 0.9376; *p* = 0.4771; genotype effect, *F*_(1,55)_ = 0.1576; *p* = 0.6929; time effect, *F*_(3.233,177.8)_ = 141.9; *p* < 0.0001; *n* = 25–32). ***C***, Maximum seizure stage reached after PTZ (30 mg/kg; 40 mg/kg dose shown in Extended Data Figure 6-1) at 16–20 months (Mann–Whitney test, *U* = 405.5; *p* = 0.3881; *n* = 30–31 per group) and ***D***, latency to each stage (two-way RM ANOVA; interaction effect, *F*_(7,413)_ = 2.161; *p* = 0.0367; genotype effect, *F*_(1,59)_ = 0.3235; *p* = 0.5716; time effect, *F*_(3.424,202.0)_ = 95.81; *p* < 0.0001; *n* = 30–31).

One measure of network hyperexcitability observed in many mouse models of AD is increased seizure susceptibility (Palop et al., 2007; Roberson et al., 2007; Minkeviciene et al., 2009). We used the PTZ seizure susceptibility assay in both young and aged mice to assess this phenotype (Löscher et al., 1991). Although the distributions appeared different in older mice, there was no significant difference in PTZ-induced seizure susceptibility, measured by max stage or latency, in young (Fig. 6*A*,*B*) or aged mice (Fig. 6*C*,*D*). Older mice of both genotypes were more susceptible to seizures than younger mice (Extended Data Fig. 6-1), so a lower dose was used for older mice.

10.1523/ENEURO.0304-25.2026.f6-1 Figure 6-1 **Both control and *Bin1*-pvKO mice show high seizure susceptibility at 16–20 months. A)** Maximum seizure stage reached after PTZ (40 mg/kg) at 16–20 months (Mann-Whitney test, U = 136, *p* = 0.1595, n = 19 per group). **B)** Probability of survival during PTZ assay (log-rank Mantel-Cox test, Chi square = 0.8383, *p* = 0.3599, n = 19 per group). Download Figure 6-1, TIF file.

**Figure 7.**
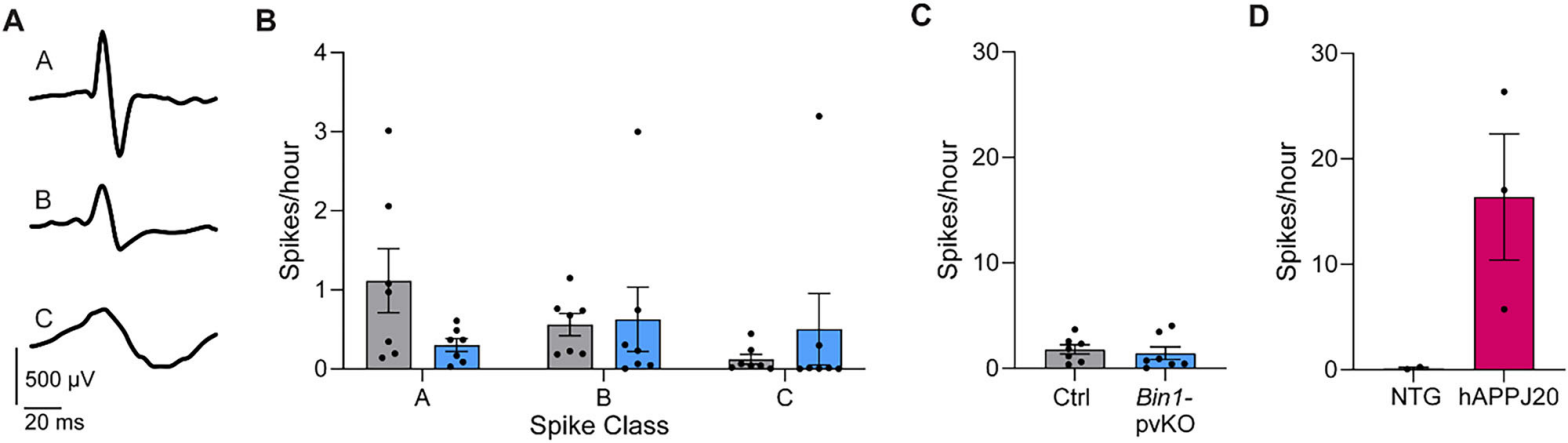
*Bin1-*pvKO mice do not show spiking like that observed in AD models. ***A***, Example waveforms used for spike classification in EEG recordings. ***B***, Spike class per hour (***A***, Welch’s *t* test, *t*_(6.477)_ = 1.968; *p* = 0.0931; ***B***, Mann–Whitney test, *U* = 18; *p* = 0.4557; ***C***, *U* = 15.50; *p* = 0.2756; *n* = 7 per group). ***C***, Total spikes per hour averaged over the entire recording (Mann–Whitney test, *U* = 19; *p* = 0.5350; *n* = 7 per group). ***D***, Spikes per hour in hAPPJ20 mice (Mann–Whitney test, *U* = 0; *p* = 0.2000; *n* = 2–3 per group).

EEG recordings are also widely used in AD mouse models to detect spontaneous epileptiform activity, which is indicative of hyperexcitability (Palop et al., 2007; Minkeviciene et al., 2009; Roberson et al., 2011; Kam et al., 2016). We recorded EEG activity from cortical electrodes continuously for 7 d. Spikes were counted and sorted into one of the three classes: A, high amplitude, quick bursting spikes; B, lower amplitude spikes with a slower return to baseline; or C, low and slow signals that passed the amplitude thresholds (Fig. 7*A*). While spiking activity varied across mice and spike classes, there were no differences in the number of spikes per hour in each class (Fig. 7*B*). Additionally, there was no difference in the average number of spikes per hour, summed across all three classes (Fig. 7*C*). As a positive control, we included hAPPJ20 mice, which are known to have epileptiform activity (Fig. 7*D*). Overall, spiking was sparse in both controls and *Bin1*-pvKO mice, showing no robust differences in network hyperexcitability in this study, especially compared with that of other AD mouse models.

### Loss of *Bin1* from PV neurons does not induce major network alterations

**Figure 8.**
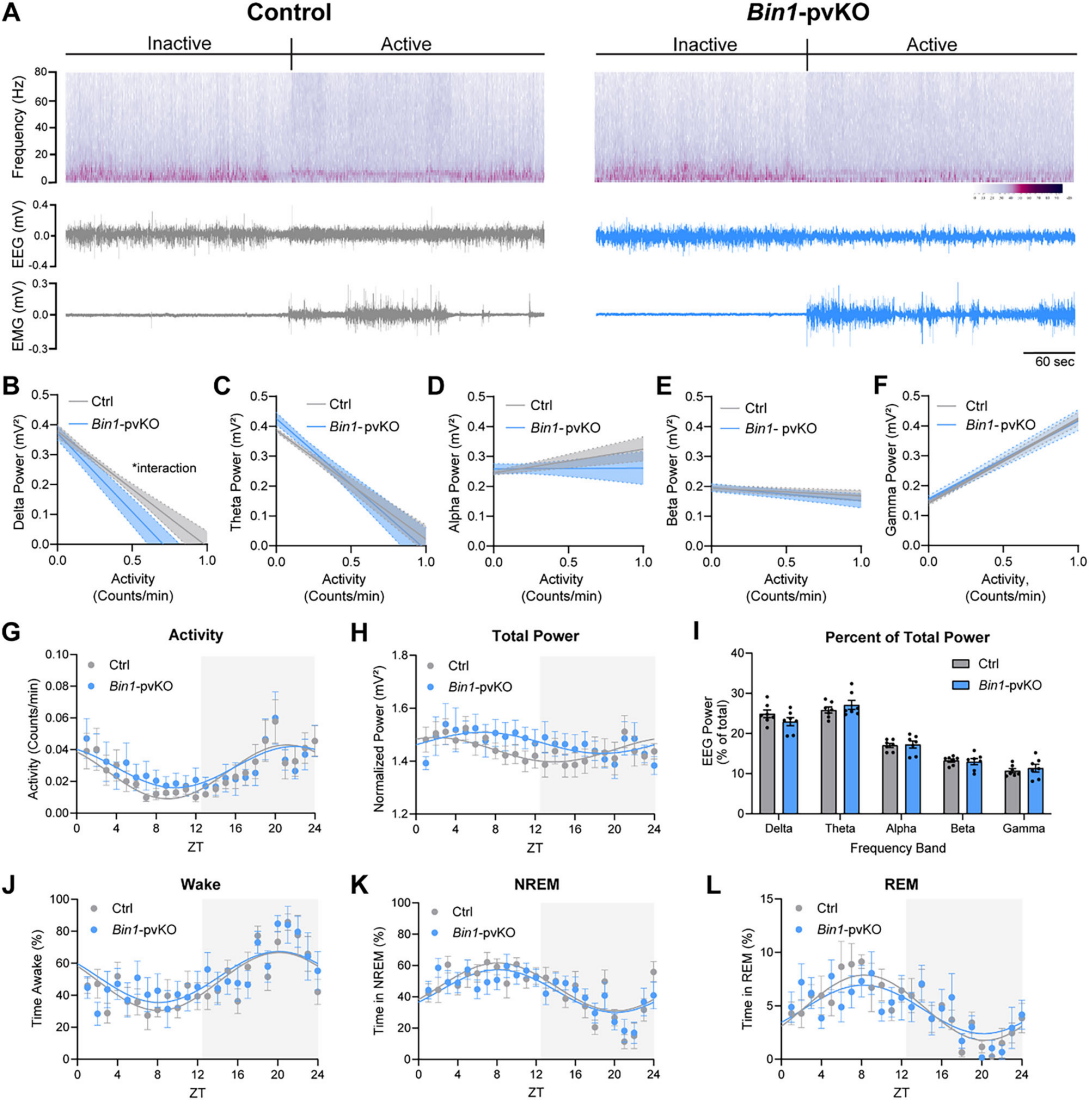
*Bin1-*pvKO mice do not show major network alterations. ***A***, Representative traces for control (left) and *Bin1-*pvKO (right) showing frequency band intensity, EEG traces, EMG traces, and locomotory activity during resting and active states. ***B***, Delta power across activity states (two-way RM ANOVA, interaction, *F*_(10,120)_ = 1.979; *p* = 0.0413; activity, *F*_(1,12)_ = 84.80; *p* < 0.0001; genotype, *F*_(1,12)_ = 2.392; *p* = 0.1479). ***C***, Theta power across activity states (two-way RM ANOVA; interaction, *F*_(10,120)_ = 0.5857; *p* = 0.8230; activity, *F*_(1,12)_ = 66.99; *p* < 0.0001; genotype, *F*_(1,12)_ = 0.1720; *p* = 0.6856). ***D***, Alpha power across activity states (two-way RM ANOVA; interaction, *F*_(10,120)_ = 1.137; *p* = 0.3404; activity, *F*_(1,12)_ = 1.315; *p* = 0.2739; genotype, *F*_(1,12)_ = 0.8494; *p* = 0.3749). ***E***, Beta power across activity states (two-way RM ANOVA; interaction, *F*_(10,120)_ = 0.5066; *p* = 0.8826; activity, *F*_(1,12)_ = 6.997; *p* = 0.0214; genotype, *F*_(1,12)_ = 0.3038; *p* = 0.5917). ***F***, Gamma power across activity states (two-way RM ANOVA, interaction, *F*_(10,120)_ = 0.2118; *p* = 0.9949; activity, *F*_(1,12)_ = 266.2; *p* < 0.0001; genotype, *F*_(1,12)_ = 0.009647; *p* = 0.9234). ***G***, Average locomotor activity over the 24-h cycle (two-way RM ANOVA; interaction, *F*_(23,276)_ = 0.353; *p* = 0.9977; time, *F*_(4.054, 48.65)_ = 6.682; *p* = 0.0002; genotype, *F*_(1,12)_ = 0.09613; *p* = 0.7618). ***H***, Average normalized total power over the 24 h cycle (two-way RM ANOVA, interaction, *F*_(23,276)_ = 1.356; *p* = 0.1316; time, *F*_(5.287, 63.44)_ = 2.795; *p* = 0.0221; genotype, *F*_(1,12)_ = 0.2240; *p* = 0.6445), individual power bands across the 24 h cycle shown in Extended Data Figure 8-1. ***I***, Distribution of percent usage of each frequency band normalized to total power (two-way RM ANOVA, interaction, *F*_(4,48)_ = 0.8896; *p* = 0.4774; frequency band, *F*_(2.584, 31.01)_ = 107.4; *p* < 0.0001; genotype, *F*_(1,12)_ = 0.2857; *p* = 0.6028). ***J***, Percent time awake over a 24 h period (two-way RM ANOVA, time effect, *F*_(7.114, 85.37)_ = 5.648; *p* < 0.0001; genotype effect, *F*_(1,12)_ = 1.494; *p* = 0.2451; interaction effect, *F*_(23,276)_ = 0.5560; *p* *=* 0.9526; *n* = 7 per group). ***K***, Percent time in NREM over a 24 h period (two-way RM ANOVA, time effect, *F*_(7.146, 85.75)_ = 5.435; *p* < 0.0001; genotype effect, *F*_(1,12)_ = 1.198; *p* = 0.2953; interaction effect, *F*_(23,276)_ = 0.5649; *p* = 0.9481; *n* = 7 per group). ***L***, Percent time in REM over a 24 h period (two-way RM ANOVA; time effect, *F*_(6.558, 78.70)_ = 4.918; *p* = 0.0002; genotype effect, *F*_(1,12)_ = 0.2486; *p* = 0.6271; interaction effect, *F*_(23,276)_ = 0.6717; *p* = 0.8720; *n* = 7 per group). Further sleep analysis shown in Extended Data Figure 8-2.

The EEG recordings also enabled us to assess how *Bin1-*pvKO affects cortical rhythms, which PV neurons regulate. We examined power spectral densities across the 7 d continuous EEG recordings, distinguishing inactive and active states by EMG and accelerometers in the transmitters (Fig. 8*A*). The raw power of each frequency band (delta, theta, alpha, beta, and gamma) was examined as a function of locomotor activity (Fig. 8*B*–*F*). As expected, delta and theta power both significantly decreased with increased activity (Fig. 8*B*,*C*), while gamma power increased with activity (Fig. 8*F*). There were no significant genotype differences in any power band, although delta power had a genotype by activity interaction, with lower power in *Bin1-*pvKO mice at higher activity levels (Fig. 8*B*).

10.1523/ENEURO.0304-25.2026.f8-1 Figure 8-1 **Individual EEG bands normalized to total power across the 24-hour cycle.** EEG power as a percentage of total power for **A)** Delta power (two-way RM ANOVA, interaction: F (23, 276) = 0.7720, *p* = 0.7653, time: F (5.751, 69.01) = 4.766, *p* = 0.0005, genotype: F (1, 12) = 2.001, *p* = 0.1826), **B)** Theta power (two-way RM ANOVA, interaction: F (23, 276) = 0.4880, *p* = 0.9785, time: F (4.513, 54.16) = 8.522, *p* < 0.0001, genotype: F (1, 12) = 0.8628, *p* = 0.3713), **C)** Alpha power (two-way RM ANOVA, interaction: F (23, 276) = 0.4113, *p* = 0.9932, time: F (5.216, 62.59) = 3.640, *p* = 0.0053, genotype: F (1, 12) = 0.04475, *p* = 0.8360), **D)** Beta power (two-way RM ANOVA, interaction: F (23, 276) = 1.476, *p* = 0.0774, time: F (3.982, 47.78) = 7.490, *p* < 0.0001, genotype: F (1, 12) = 0.1253, *p* = 0.7295), and **E)** Gamma power (two-way RM ANOVA, interaction: F (23, 276) = 0.8836, *p* = 0.6210, time: F (5.688, 68.25) = 8.691, *p* < 0.0001, genotype: F (1, 12) = 0.3647, *p* = 0.5571). **F)** 24-hour delta:theta ratio (two-way RM ANOVA, interaction: F (23, 276) = 1.175, *p* = 0.2666, time: F (3.879, 46.55) = 1.648, *p* = 0.1797, genotype: F (1, 12) = 2.823, *p* = 0.1187). Download Figure 8-1, TIF file.

10.1523/ENEURO.0304-25.2026.f8-2 Figure 8-2 ***Bin1-*pvKO mice show no overt sleep differences. A)** Representative traces of EEG and EMG signal during wake, NREM, and REM states. **B)** Average number of sleep bouts (unpaired t test, t (12) = 0.09563, *p* = 0.9254). **C)** Average sleep bout duration (unpaired t test, t (12) = 0.4459, *p* = 0.6636). **D)** Average percent time awake during day, night, and over the 24-hour cycle (two-way RM ANOVA, time effect: F (1.678, 20.13) = 643.8, *p* < 0.0001, genotype effect: F (1, 12) = 1.677, *p* = 0.2196, interaction effect: F (2, 24) = 0.6500, *p* = 0.5310, n = 7 per group). **E)** Average percent time in NREM during day, night, and over the 24-hour cycle (two-way RM ANOVA, time effect: F (1.864, 22.37) = 590.1, *p* < 0.0001, genotype effect: F (1, 12) = 1.351, *p* = 0.2677, interaction effect: F (2, 24) = 0.6686, *p* = 0.5217, n = 7 per group). **F)** Average percent time in REM during day, night, and over the 24-hour cycle (two-way RM ANOVA, time effect: F (1.686, 20.24) = 291.8, *p* < 0.0001, genotype effect: F (1, 12) = 0.2187, *p* = 0.6484, interaction effect: F (2, 24) = 0.4309, *p* = 0.6548, n = 7 per group). **G)** Power during wake state (two-way RM ANOVA, frequency effect: F (1, 24) = 15.78, *p* = 0.0006; genotype effect: F (3, 24) = 0.6865, *p* = 0.5691, interaction effect: F (120, 960) = 0.6761, *p* = 0.9963, n = 7 per group). **H)** Power during NREM state during lights on and off (two-way RM ANOVA, frequency effect: F (1.001, 24.04) = 4.732, *p* = 0.0396; genotype effect: F (3, 24) = 1.283, *p* = 0.3028; interaction effect: F (120, 960) = 1.321, *p* = 0.0160; n = 7 per group). **I)** Power during REM state during lights on and off (two-way RM ANOVA, frequency effect: F (1.000, 24.01) = 1.684, *p* = 0.2068; genotype effect: F (3, 24) = 0.8456, *p* = 0.4825; interaction effect: F (120, 960) = 0.8313, *p* = 0.8998, n = 7 per group). Download Figure 8-2, TIF file.

Locomotor activity was rhythmic across the circadian cycle, as expected (Fig. 8*G*). Total EEG power (ranging from 0.5 to 80 Hz) was also rhythmic across the circadian cycle and not different between genotypes (Fig. 8*H*). Additionally, the contribution of each frequency band to total power was not different between groups (Fig. 8*I*; Extended Data Fig. 8-1). Finally, we explored sleep phenotypes in the recordings given the potential differences in delta power, which increases during sleep. The percentage of time spent in each sleep stage, sleep disruption, and power during each sleep stage was not different between groups (Fig. 8*J*–*L*; Extended Data Fig. 8-2). Overall, *Bin1-*pvKO mice showed a slight reduction of delta power at higher activity levels but minimal overall network alterations.

## Discussion

In this study, we investigated the effects of *Bin1* loss from PV neurons, particularly on cognition and network excitability phenotypes that are abnormal in AD mouse models. *Bin1*-pvKO mice had normal gross brain architecture and general health (Fig. 2) and normal performance on most behavioral tests including measures of learning and memory (Figs. 3–5), with only some decreased exploratory behavior with aging (Fig. 3). *Bin1*-pvKO mice did not show increased seizure susceptibility (Fig. 6) or increased spiking on EEG (Fig. 7). Power spectral analysis of EEG recordings was also mostly normal, except for some modest reduction in delta power (Fig. 8). Overall, loss of *Bin1* from PV neurons did not cause obvious cognitive dysfunction or network hyperexcitability and did not phenocopy AD models.

These data help refine the potential roles of BIN1 in inhibitory neurons. In addition to the literature cited in the Introduction that motivated this study, new evidence supports an important role of BIN1 in inhibitory neurons (Barata et al., 2025). In cultured mouse neurons, Bin1 was predominantly localized at inhibitory synapses, and either Bin1 knockdown or expression of AD-associated Bin1 variants reduced inhibitory synapses and induced hyperexcitability (Barata et al., 2025). Our data suggest that in vivo, PV neurons are unlikely to be the site of these effects, pointing instead to other inhibitory neuron types. In the cortex, PV neurons are the most abundant type of interneuron, followed by those expressing somatostatin (SST) and vasoactive intestinal peptide (VIP; Swanson and Maffei, 2019). While PV neurons are fast-spiking with perisomatic projections that help to synchronize network activity (Hu et al., 2014), SST neurons have slower spiking, dendritic projections, and a larger role in learning and plasticity (Swanson and Maffei, 2019). Interestingly, a recent multiomic quantitative neuropathology study identified loss of SST neurons as an early event in AD, whereas the loss of excitatory neurons and PV and VIP neurons occurred in later stages (Gabitto et al., 2024). Future studies should examine BIN1 effects in SST neurons, either alone or in combination with PV neurons.

The reduced delta power at higher activity levels in *Bin1*-pvKO mice (Fig. 8*B*) was somewhat surprising, since PV neurons are more closely associated with gamma power (Hu et al., 2014; Antonoudiou et al., 2020), which was unchanged. However, there are links between PV neurons and delta. PV neuron firing is phase-locked to delta oscillations (Yao et al., 2020), and PV disruption leads to decreased delta power (Kuki et al., 2015; Ognjanovski et al., 2017). This is likely an indirect effect of aberrant circuit excitability, with inhibition being altered by PV dysfunction.

While we identified no major abnormalities in *Bin1*-pvKO mice, the study design had several strengths. One was the sensitive and rigorous outcome measures evaluated. In addition to traditional behavioral methods, we utilized unbiased, machine learning–based behavioral profiling to identify phenotypes not apparent with traditional behavior methods (Gschwind et al., 2023; Miller et al., 2024). Additionally, the week-long continuous EEG recordings provided data throughout the circadian cycle and across activity states and sleep stages. Finally, with the large number of mice tested (152 control vs 134 *Bin1*-pvKO mice, across multiple cohorts at different ages for various outcome measures, as detailed in Materials and Methods), we had ample power to detect medium-to-large effects.

This study also has important limitations and caveats. First, while the median expression of *Bin1* mRNA in PV cells was zero in *Bin1*-pvKO mice, with an average 75% reduction, there was still a small population with some *Bin1* present (Fig. 1*B*), and it is possible that the remaining *Bin1-*positive PV cells could compensate for those without *Bin1.* Second, although we had a large sample size for most experiments, we were limited in the number of mice for power spectral density analysis since the resource-intensive nature of the EEG experiments precluded running analysis in multiple cohorts. Additionally, these recordings were cortical and would likely not detect abnormalities localized to subcortical regions. Third, while our data indicate that *Bin1* loss in PV neurons is not sufficient to mimic effects seen in AD models, they do not exclude the role of BIN1 in PV neurons in AD, which may require a “second hit,” such as expression of human Aβ, or cooperative effects with other cell types. Future studies could further explore BIN1’s role in the context of AD models. Lastly, while we focused on major outcome measures that have been studied in AD models, it is always possible that *Bin1*-pvKO mice have other abnormalities that we did not examine. For example, we did not examine the intrinsic properties of PV neurons at the single-cell level by patch electrophysiology.

In summary, *Bin1*-pvKO mice showed no obvious changes in cognition or network excitability despite substantial Bin1 loss in parvalbumin neurons. These findings are informative given the growing evidence for the important role of BIN1 in inhibitory neurons and suggest that inhibitory neuron classes beyond PV likely contribute. Specifically, SST neurons, which are also vulnerable in AD, may contribute to BIN1-related effects on neural circuits. Future studies should examine the role of BIN1 in SST neurons, either alone or in combination with PV, in AD-related effects on cognition and network excitability.

## Synthesis

Reviewing Editor: Harald Sontheimer, University of Virginia Decisions are customarily a result of the Reviewing Editor and the peer reviewers coming together and discussing their recommendations until a consensus is reached. When revisions are invited, a fact-based synthesis statement explaining their decision and outlining what is needed to prepare a revision will be listed below. The following reviewer(s) agreed to reveal their identity: Bhanu Tewari.

The revised manuscript provides explicit evidence of the Bin 1 knockout in PV neurons, which previously was the primary concern.
