# Supplementary material for: Parvalbumin Neuron–Targeted Loss of Alzheimer’s Disease Risk Gene BIN1 Is Insufficient to Drive Cognitive or Network Excitability Changes
Source: eNeuro. 2026 Mar 25;13(3):ENEURO.0304-25.2026. doi: 10.1523/ENEURO.0304-25.2026 (PMC13064429; doi:10.1523/ENEURO.0304-25.2026)
Supplement: Data 1 — Statistics table. Organized by Figure panel, showing description, test, n, relevant statistics, p-values, means, and standard errors. Download Data 1, DOCX file. [file eneuro-13-ENEURO.0304-25.2026-s002.docx]

| **Figure** | **Description** | **Test** | **n (per group)** | **Statistic (df)** | **p-value** | **Mean/Estimate ± SEM** |
| --- | --- | --- | --- | --- | --- | --- |
| 1B | Percent area *Bin1* in PV cells | Linear mixed-effects model (Mean ~ Genotype + 1\|mouse) | Mice = 3, 2  Cells = 1149, 523 | *t (*3) = −5.47 | *p* = 0.012 | *β =* −1.42, *SE =* 0.26, |
| 1C | Percent area *Bin1* in Gad cells | Linear mixed-effects model (Mean ~ Genotype + 1\|mouse) | Mice = 3, 2  Cells = 1617, 927 | *t (*3) = −0.699 | *p* = 0.5346 | *β =* −0.23, *SE =* 0.33 |
| 1-1D | Bin1 protein levels in cortex | Unpaired t test | 7/group | t (12) = 2.302 | *p* = 0.0400 | Ctrl: 1.000 ± 0.03476  KO: 0.8508 ± 0.05468 |
| 2B | Probability of survival | Mantel-Cox | Ctrl: 63 M, 71 F  KO: 70 M, 66 F | Χ^2^ (3) = 1.330 | *p* = 0.7221 |  |
| 2C | Weight (g) young mice | Two-way ANOVA | Ctrl: 47 M, 40 F  KO: 44 M, 31 F | Interaction: F (1, 158) = 0.1807  Sex: F (1, 158) = 19.83  Genotype: F (1, 158) = 0.2965 | Interaction: *p* = 0.6713  Sex: *p* < 0.0001  Genotype: *p* = 0.5868 | Ctrl Males: 33.134 ± 1.088  Ctrl Females: 27.080 ± 1.818  KO Males: 32.955 ± 1.402  KO Females: 25.623 ± 1.693 |
| 2D | Weight (g) aged mice | Two-way ANOVA | Ctrl: 28 M, 34 F  KO: 31 M, 21 F | Interaction: F (1, 110) = 2.447  Sex: F (1, 110) = 14.86  Genotype: F (1, 110) = 1.745 | Interaction: *p* = 0.1206  Sex: *p =* 0.0002  Genotype: *p* = 0.1892 | Ctrl Males: 39.996 ± 1.720  Ctrl Females: 31.094 ± 0.934  KO Males: 39.597 ± 1.407  KO Females: 35.833 ± 2.730 |
| 2B | Probability of survival | Uncorrected Fisher’s LSD test for multiple comparisons | Ctrl: 63 M, 71 F  KO: 70 M, 66 F | Females: t (110) = 1.979  Males: t (110) = 0.1777  Ctrl: t (110) = 4.044  KO: t (110) = 1.544 | Females: *p =* 0.0503  Males: *p* = 0.8593  Ctrl: *p* < 0.0001  KO: *p* = 0.1256 |  |
| 3A | Aged Y Maze arm entrances | Unpaired t test | Ctrl: 35  KO: 31 | t (64) = 2.247 | *p =* 0.0281 | Ctrl: 24.34 ± 1.097  KO: 20.55 ± 1.298 |
| 3B  3C | Aged Y Maze Distance traveled  Aged EPM distance traveled | Unpaired t test | Ctrl: 35  KO: 31  Ctrl: 16  KO: 12 | t (64) = 2.220 | *p =* 0.0300 | Ctrl: 1552 ± 64.88  KO: 1322 ± 82.29 |
|  |  | Unpaired t test |  | t (26) = 1.130 | *p =* 0.2688 | Ctrl: 1055 ± 76.73  KO: 917 ± 95.31 |
| 3D | Aged Open Field distance traveled by minute | Two-way RM ANOVA | Ctrl: 35  KO: 31 | Interaction: F (9, 576) = 0.3908  Time: F (1.808, 115.7) = 61.76  Genotype: F (1, 64) = 0.5156 | Interaction: *p* = 0.9397  Time: *p <* 0.0001  Genotype: *p* = 0.4753 | Ctrl: 299.3  KO: 276.7  SE: 31.51 |
| 3E | Aged EPM % Open Arms | Mann-Whitney test | Ctrl: 35  KO: 31 | U = 506 | *p* = 0.6435 | Ctrl: 34.20 ± 3.553  KO: 32.99 ± 4.822 |
| 3F | Aged Open Field Time in Center | Mann-Whitney test | Ctrl: 35  KO: 31 | U = 541 | *p* = 0.9872 | Ctrl: 9.471 ± 1.098  KO: 9.615 ± 1.204 |
| 3G | Aged Y Maze % Alternations | Mann-Whitney test | Ctrl: 35  KO: 30 | U = 452.5 | *p* = 0.3432 | Ctrl: 50.50 ± 1.850  KO: 53.17 ± 2.434 |
| 3H | Aged FC Time Freezing | Two-way ANOVA | Ctrl: 34  KO: 31 | Interaction: F (2, 126) = 0.8953  Time: F (1.736, 109.3) = 298.0  Genotype: F (1, 63) = 0.4092 | Interaction: *p* = 0.4111  Time: *p <* 0.0001  Genotype: *p* = 0.5247 | Ctrl: Baseline 2.354 ± 0.582, PC 62.285 ±3.197 Test 31.147 ±2.640  KO: Baseline 4.169 ±1.449, PC 60.607 ±1.449, Test 35.839 ±3.005 |
| 3-1A | Y Maze Entrances by sex | Two-way ANOVA | Ctrl: 14 M, 21 F  KO: 21 M, 10 F | Interaction: F (1, 62) = 0.05641  Sex: F (1, 62) = 12.51  Genotype: F (1, 62) = 1.848 | Interaction: *p =* 0.8131  Sex: *p =* 0.0008  Genotype: *p =* 0.1789 | Ctrl Males: 20.643 ± 1.417  Ctrl Females: 26.810 ± 1.334  KO Males: 18.810 ± 1.561  KO Females: 24.200 ± 1.965 |
| 3-1B | Y Maze Distance Traveled by sex | Two-way ANOVA | Ctrl: 14 M, 21 F  KO: 21 M, 10 F | Interaction: F (1, 62) = 0.0002152  Sex: F (1, 62) = 9.577  Genotype: F (1, 62) = 1.923 | Interaction: *p =* 0.9883  Sex: *p =* 0.0030  Genotype: *p =* 0.1705 | Ctrl Males: 1360.697 ± 82.995  Ctrl Females: 1679.522 ± 83.113  KO Males: 1220.009 ± 99.592  KO Females: 1535.826 ± 127.230 |
| 4A | Young Y Maze Arm Entrances | Unpaired t test | Ctrl: 34  KO: 31 | t (63) = 0.1412 | *p* = 0.8882 | Ctrl: 35.71 ± 1.510  KO: 35.39 ± 1.688 |
| 4B | Young Open Field Distance Traveled by minute | Two-way RM ANOVA | Ctrl: 34  KO: 30 | Interaction: F (9, 558) = 0.5879, Genotype: F (1, 62) = 0.1393  Time: F (3.383, 209.7) = 56.91 | Interaction: *p* = 0.8075, Genotype: *p* = 0.7103, Time: *p* < 0.0001 | Ctrl: 223.0  KO: 216.8  SE: 16.58 |
| 4C | Young Open Field Time in Center | Unpaired t test | Ctrl: 20  KO: 18 | t (36) = 1.098 | *p* = 0.2794 | Ctrl: 9.455 ± 1.050  KO: 8.026 ± 0.7194 |
| 4D | Young EPM % Time in Open Arms | Unpaired t test | Ctrl: 32  KO: 29 | t (59) = 0.5556 | *p* = 0.5806 | Ctrl: 38.49 ± 3.242  KO: 35.99 ± 3.079 |
| 4E | Young Y Maze Arm Alternations | Unpaired t test | Ctrl: 34  KO: 31 | t (63) = 0.1895 | *p* = 0.8503 | Ctrl: 54.78 ± 1.541  KO: 55.28 ± 2.180 |
| 4F | Young FC Time Freezing | Two-way ANOVA | Ctrl: 33  KO: 29 | Interaction: F (2, 120) = 1.651, Genotype effect: F (1, 60) = 1.484  Time: F (1.985, 119.1) = 143.2 | Interaction: *p* = 0.1961, Genotype: *p* = 0.2280, Time: *p* < 0.0001 | Ctrl: Baseline 2.277 ± 0.762, PC 42.659 ±3.466 Test 25.515 ±2.807  KO: Baseline 3.549 ±1.790, PC 44.240 ±3.876, Test 34.621 ±4.357 |
| 5A | Syllable usage norm to Ctrl | Two-way RM ANOVA | 24/group | Interaction: F (36, 1656) = 0.5730,  Genotype: F (1, 46) = 0.004145, Syllable: F (5.837, 268.5) = 0.5730 | Interaction: *p* = 0.9806, Genotype: *p* = 0.9489, Syllable: *p =* 0.7471 | Ctrl: 1.000  KO: 1.003  SE: 0.03963 |
| 5B | Grouped syllable usage time | Two-way RM ANOVA | 24/group | Interaction: F (6, 276) = 0.4985  Genotype: F (1, 46) = 0.02772, Syllable: F (1.960, 90.16) = 349.1 | Interaction: *p* = 0.8093, Genotype: *p =* 0.8685, Syllable: *p* < 0.0001 | Ctrl: 12.53  KO: 12.55  SE: 0.07244 |
| 5C | Distance Traveled | Unpaired t test | 24/group | t (46) = 0.7510 | *p* = 0.4565 | Ctrl: 16133 ± 546.4  KO: 15490 ± 659.7 |
| 6A | Young PTZ Max Stage | Mann-Whitney test | Ctrl: 32  KO: 25 | U = 371.5 | *p* = 0.6426 | Ctrl: 3.125 ± 0.3803  KO: 3.440 ± 0.4693 |
| 6B | Young PTZ Latency | two-way RM ANOVA | Ctrl: 32  KO: 25 | Interaction: F (7, 385) = 0.9376 Genotype: F (1, 55) = 0.1576  Time: F (3.233, 177.8) = 141.9 | Interaction: *p* = 0.4771, Genotype: *p* = 0.6929,  Time: *p* < 0.0001 | Ctrl: 13.66  KO: 14.12  SE: 1.149 |
| 6C | Aged PTZ Max Stage | Mann-Whitney test | Ctrl: 31  KO: 30 | U = 405.5 | *p* = 0.3881 | Ctrl: 3.452 ± 0.3875  KO: 4.267 ± 0.5162 |
| 6D | Aged PTZ Latency | two-way RM ANOVA | Ctrl: 31  KO: 30 | Interaction: F (7, 413) = 2.161  Genotype: F (1, 59) = 0.3235  Time: F (3.424, 202.0) = 95.81 | Interaction: *p* = 0.0367, Genotype: *p* = 0.5716, Time: *p* < 0.0001 | Ctrl: 12.68  KO: 11.89  SE: 1.386 |
| 6-1A | Aged PTZ Max Stage at 40 mg/kg | Mann-Whitney test | 19/group | U = 136 | *p* = 0.1595 | Ctrl: 5.895 ± 0.4950  KO: 6.842 ± 0.3993 |
| 6-1B | Aged PTZ Survival at 40 mg/kg | log-rank Mantel-Cox test | 19/group | Χ^2^ (1) = 0.8383 | *p* = 0.3599 |  |
| 7B | Spike class per hour | A: Welch’s t test  B: Mann-Whitney test  C: Mann-Whitney test | 7/group | A: t (6.477) = 1.968  B: U = 18  C: U = 15.50 | A: *p* = 0.0931;  B: *p* = 0.4557;  C: *p* = 0.2756 | A: 187.4 ±68.06; 50.86 ±13.58  B: 94.29 ±23.51, 105.6 ±68.30  C: 20.57 ±10.34, 84.57 ±75.72 |
| 7C | Total spikes per hour | Mann-Whitney test | 7/group | U = 19 | *p* = 0.5350 | Ctrl: 1.799 ± 0.4457  KO: 1.435 ± 0.6092 |
| 7D | hAPPJ20 spikes per hour | Mann-Whitney test | Ctrl: 2  KO: 3 | U = 0 | *p* = 0.2000 | NTG: 0.1569 ± 0.09620  J20: 16.38 ± 5.966 |
| 8B | Delta power x Activity | Two-way RM ANOVA | 7/group | Interaction: F (10, 120) = 1.979, Activity: F (1, 12) = 84.80, Genotype: F (1, 12) = 2.392 | Interaction: *p* = 0.0413, Activity: *p* < 0.0001, Genotype: *p* = 0.1479 | Ctrl: -0.008457  KO: -0.15445  SE: 0.09441 |
| 8C | Theta power x Activity | Two-way RM ANOVA | 7/group | Interaction: F (10, 120) = 0.5857, Activity: F (1, 12) = 66.99, Genotype: F (1, 12) = 0.1720 | Interaction: *p* = 0.8230, Activity: *p* < 0.0001, Genotype: *p* = 0.6856 | Ctrl: 0.02234  KO: -0.01366  SE: 0.08680 |
| 8D | Alpha power x Activity | Two-way RM ANOVA | 7/group | Interaction: F (10, 120) = 1.137, Activity: F (1, 12) = 1.315, Genotype: F (1, 12) = 0.8494 | Interaction: *p* = 0.3404, Activity: *p* = 0.2739, Genotype: *p* = 0.3749 | Ctrl: 0.3239  KO: 0.2610  SE: 0.06829 |
| 8E | Beta power x Activity | Two-way RM ANOVA | 7/group | Interaction: F (10, 120) = 0.5066  Activity: F (1, 12) = 6.997 Genotype: F (1, 12) = 0.3038 | Interaction: *p* = 0.8826, Activity: *p* = 0.0214, Genotype: *p* = 0.5917 | Ctrl: 0.1685  KO: 0.1518  SE: 0.03035 |
| 8F | Gamma power x Activity | Two-way RM ANOVA | 7/group | Interaction: F (10, 120) = 0.2118 Activity: F (1, 12) = 266.2  Genotype: F (1, 12) = 0.009647 | Interaction: *p* = 0.9949, Activity: *p* < 0.0001, Genotype: *p* = 0.9234 | Ctrl: 0.4230  KO: 0.4189  SE: 0.04131 |
| 8G | 24-hour Activity | Two-way RM ANOVA | 7/group | Interaction: F (23, 276) = 0.353  Time: F (4.054, 48.65) = 6.682  Genotype: F (1, 12) = 0.09613 | Interaction: *p* = 0.9977, Time: *p* = 0.0002, Genotype: *p* = 0.7618 | Ctrl: 0.02619  KO: 0.02869  SE: 0.008084 |
| 8H | 24-hour Total Power | Two-way RM ANOVA | 7/group | Interaction: F (23, 276) = 1.356  Time: F (5.287, 63.44) = 2.795  Genotype: F (1, 12) = 0.2240 | Interaction: *p* = 0.1316,  Time: *p* = 0.0221, Genotype: *p* = 0.6445 | Ctrl: 1.443  KO: 1.469  SE: 0.05546 |
| 8I | Frequency band distribution of total power | Two-way RM ANOVA | 7/group | Interaction: F (4, 48) = 0.8896  Frequency band: F (2.584, 31.01) = 107.4 Genotype: F (1, 12) = 0.2857 | Interaction: p = 0.4774, Frequency band: p < 0.0001, Genotype: p = 0.6028 | Ctrl: 18.33  KO: 18.30  SE: 0.04935 |
| 8J | 24-hour Time Awake | Two-way RM ANOVA | 7/group | Interaction: F (23, 276) = 0.5560,  Time: F (7.114, 85.37) = 5.648, Genotype: F (1, 12) = 1.494 | Interaction: *p =* 0.9526  Time: *p* < 0.0001, Genotype: *p* = 0.2451 | Ctrl: 48.60  KO: 51.41  SE: 2.292 |
| 8K | 24-hour Time in NREM | Two-way RM ANOVA | 7/group | Interaction: F (23, 276) = 0.5649, Time: F (7.146, 85.75) = 5.435, Genotype: F (1, 12) = 1.198 | Interaction: *p* = 0.9481  Time: *p* < 0.0001, Genotype: *p* = 0.2953 | Ctrl: 46.32  KO: 43.69  SE: 2.399 |
| 8L | 24-hour Time in REM | Two-way RM ANOVA | 7/group | Interaction: F (23, 276) = 0.6717,  Time: F (6.558, 78.70) = 4.918, Genotype: F (1, 12) = 0.2486 | Interaction: *p* = 0.8720  Time: *p* = 0.0002, Genotype: *p* = 0.6271, | Ctrl: 4.825  KO: 4.666  SE: 0.3184 |
| 8-1A | 24-hour Delta norm to total | Two-way RM ANOVA | 7/group | Interaction: F (23, 276) = 0.7720  Time: F (5.751, 69.01) = 4.766  Genotype: F (1, 12) = 2.001 | Interaction: *p* = 0.7653,  Time: *p* = 0.0005  Genotype: *p* = 0.1826 | Ctrl: 24.87  KO: 22.92  SE: 13.80 |
| 8-1B | 24-hour Theta norm to total | Two-way RM ANOVA | 7/group | Interaction: F (23, 276) = 0.4880  Time: F (4.513, 54.16) = 8.522  Genotype: F (1, 12) = 0.8628 | Interaction: *p* = 0.9785,  Time: *p* < 0.0001  Genotype: *p* = 0.3713 | Ctrl: 25.83  KO: 27.08  SE: 13.46 |
| 8-1C | 24-hour Alpha norm to total | Two-way RM ANOVA | 7/group | Interaction: F (23, 276) = 0.4113  Time: F (5.216, 62.59) = 3.640  Genotype: F (1, 12) = 0.04475 | Interaction: *p* = 0.9932,  Time: *p* = 0.0053  Genotype: *p* = 0.8360 | Ctrl: 17.01  KO: 17.22  SE: 10.26 |
| 8-1D | 24-hour Beta norm to total | Two-way RM ANOVA | 7/group | Interaction: F (23, 276) = 1.476  Time F (3.982, 47.78) = 7.490  Genotype: F (1, 12) = 0.1253 | Interaction: *p* = 0.0774,  Time: *p* < 0.0001  Genotype: *p* = 0.7295 | Ctrl: 13.26  KO: 12.96  SE: 0.8593 |
| 8-1E | 24-hour Gamma norm to total | Two-way RM ANOVA | 7/group | Interaction: F (23, 276) = 0.8836  Time: F (5.688, 68.25) = 8.691  Genotype: F (1, 12) = 0.3647 | Interaction: *p* = 0.6210,  Time: *p* < 0.0001  Genotype: *p* = 0.5571 | Ctrl: 10.69  KO: 11.34  SE: 1.087 |
| 8-1F | 24-hour Delta:Theta ratio | Two-way RM ANOVA | 7/group | Interaction: F (23, 276) = 1.175  Time: F (3.879, 46.55) = 1.648  Genotype: F (1, 12) = 2.823 | Interaction: *p* = 0.2666,  Time: *p* = 0.1797  Genotype: *p* = 0.1187 | Ctrl: 0.9687  KO: 0.8542  SE: 0.06815 |
| 8-2B | Number of sleep bouts | Unpaired t test | 7/group | t (12) = 0.09563 | *p* = 0.9254 | Ctrl: 298.6 ± 14.07  KO: 301.4 ± 26.35 |
| 8-2C | Sleep bout duration | Unpaired t test | 7/group | t (12) = 0.4459 | *p* = 0.6636 | Ctrl: 148.3 ± 7.734  KO: 142.6 ± 10.25 |
| 8-2D | Grouped Time Awake | Two-way RM ANOVA | 7/group | Interaction: F (2, 24) = 0.6500  Time: F (1.678, 20.13) = 643.8  Genotype: F (1, 12) = 1.677 | Interaction: *p* = 0.5310,  Time: *p* < 0.0001  Genotype: *p* = 0.2196 | Ctrl: 32.40  KO: 34.36  SE: 1.513 |
| 8-2E | Grouped Time in NREM | Two-way RM ANOVA | 7/group | Interaction: F (2, 24) = 0.6686  Time: F (1.864, 22.37) = 590.1  Genotype: F (1, 12) = 1.351 | Interaction: *p* = 0.5217,  Time: *p* < 0.0001  Genotype: *p* = 0.2677 | Ctrl: 30.88  KO: 29.03  SE: 1.589 |
| 8-2F | Grouped Time in REM | Two-way RM ANOVA | 7/group | Interaction: F (2, 24) = 0.4309  Time: F (1.686, 20.24) = 291.8  Genotype: F (1, 12) = 0.2187 | Interaction: *p* = 0.6548,  Time: *p* < 0.0001  Genotype: *p* = 0.6484 | Ctrl: 3.216  KO: 3.116  SE: 0.2145 |
| 8-2G | Power during wake | Two-way RM ANOVA | 7/group | Interaction: F (120, 960) = 0.6761  Frequency: F (1, 24) = 15.78  Genotype: F (3, 24) = 0.6865 | Interaction: *p* = 0.9963,  Frequency: *p* = 0.0006  Genotype: *p* = 0.5691 | Lights on:  Ctrl: 6.956 ±6.412  KO: 4.033 ±3.678  Lights off:  Ctrl: 9.873 ±9.200  KO: 4.900 ±4.469 |
| 8-2H | Power during NREM | Two-way RM ANOVA | 7/group | Interaction: F (120, 960) = 1.321  Frequency: F (1.001, 24.04) = 4.732  Genotype: F (3, 24) = 1.283 | Interaction: *p* = 0.0160,  Frequency: *p* = 0.0396  Genotype: *p* = 0.3028 | Lights on:  Ctrl: 0.1455 ±0.1180  KO: 0.1047 ±0.06595  Lights off:  Ctrl: 0.3879 ±0.3268  KO: 0.04330 ±0.0123 |
| 8-2I | Power during REM | Two-way RM ANOVA | 7/group | Interaction: F (120, 960) = 0.8313  Frequency: F (1.000, 24.01) = 1.684  Genotype: F (3, 24) = 0.8456 | Interaction: *p* = 0.8998,  Frequency: *p* = 0.2068  Genotype: *p* = 0.4825 | Lights on:  Ctrl: 0.1809 ±0.1633  KO: 0.01440 ±0.002694  Lights off:  Ctrl: 0.5535 ±0.4991  KO: 0.03444 ±0.006094 |
