# Supplementary figures and images for: Parvalbumin Neuron–Targeted Loss of Alzheimer’s Disease Risk Gene BIN1 Is Insufficient to Drive Cognitive or Network Excitability Changes
Source: eNeuro. 2026 Mar 25;13(3):ENEURO.0304-25.2026. doi: 10.1523/ENEURO.0304-25.2026 (PMC13064429; doi:10.1523/ENEURO.0304-25.2026)

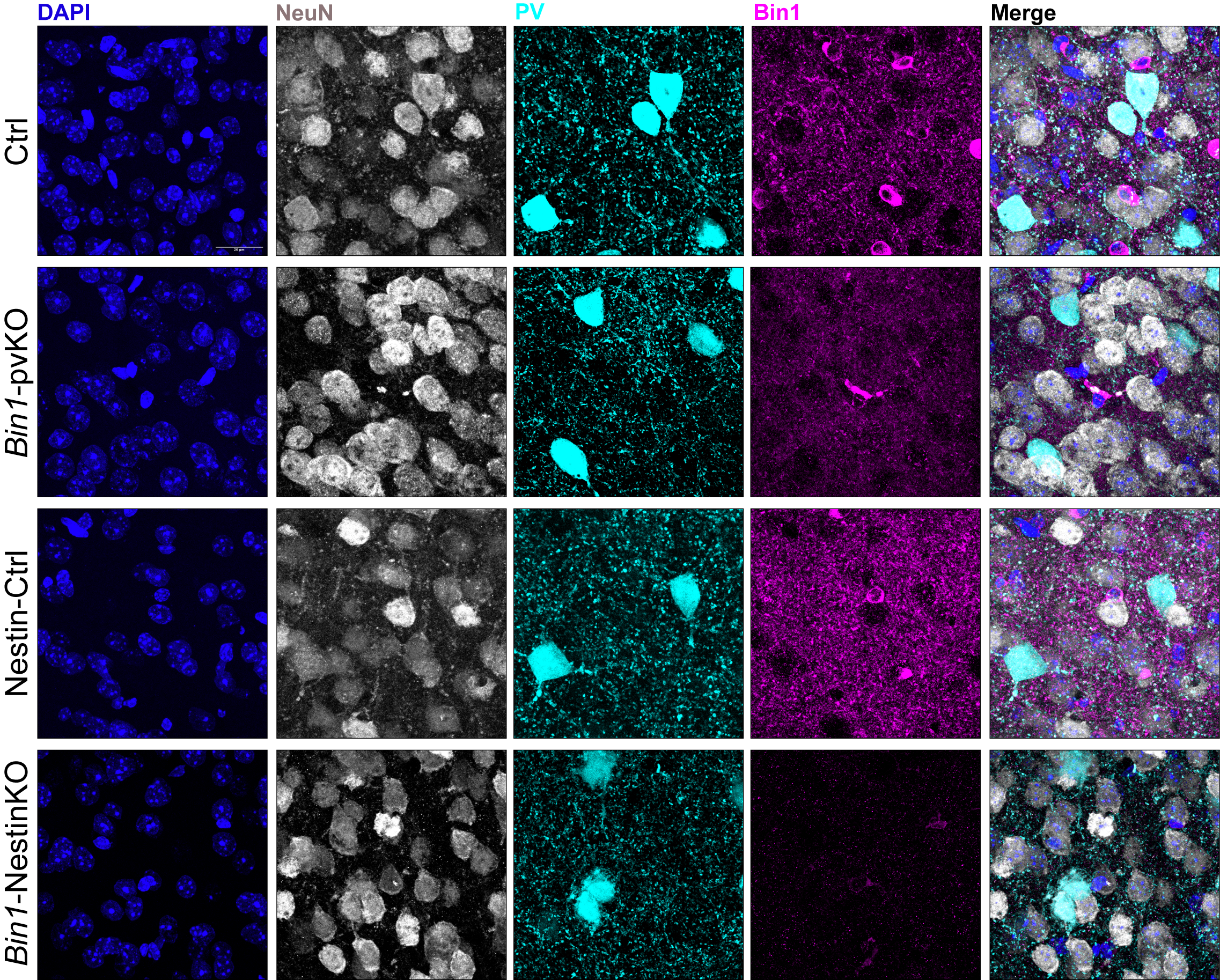

Supplement: Figure 1-1 — Bin1 immunohistochemistry in Bin1-pvKO mice. Representative immunofluorescence images from the cortex of Ctrl, Bin1-pvKO, Nestin-Ctrl, and Bin1-NestinKO mice showing DAPI (blue), NeuN (gray), PV (cyan), and Bin1 (magenta) signals individually and merged (aged 5–7 months). These are maximum intensity projections from the z-stack images used to generate the 3D reconstructions in Fig. 1D. Download Figure 1-1, TIF file. [file eneuro-13-ENEURO.0304-25.2026-s003.tif]

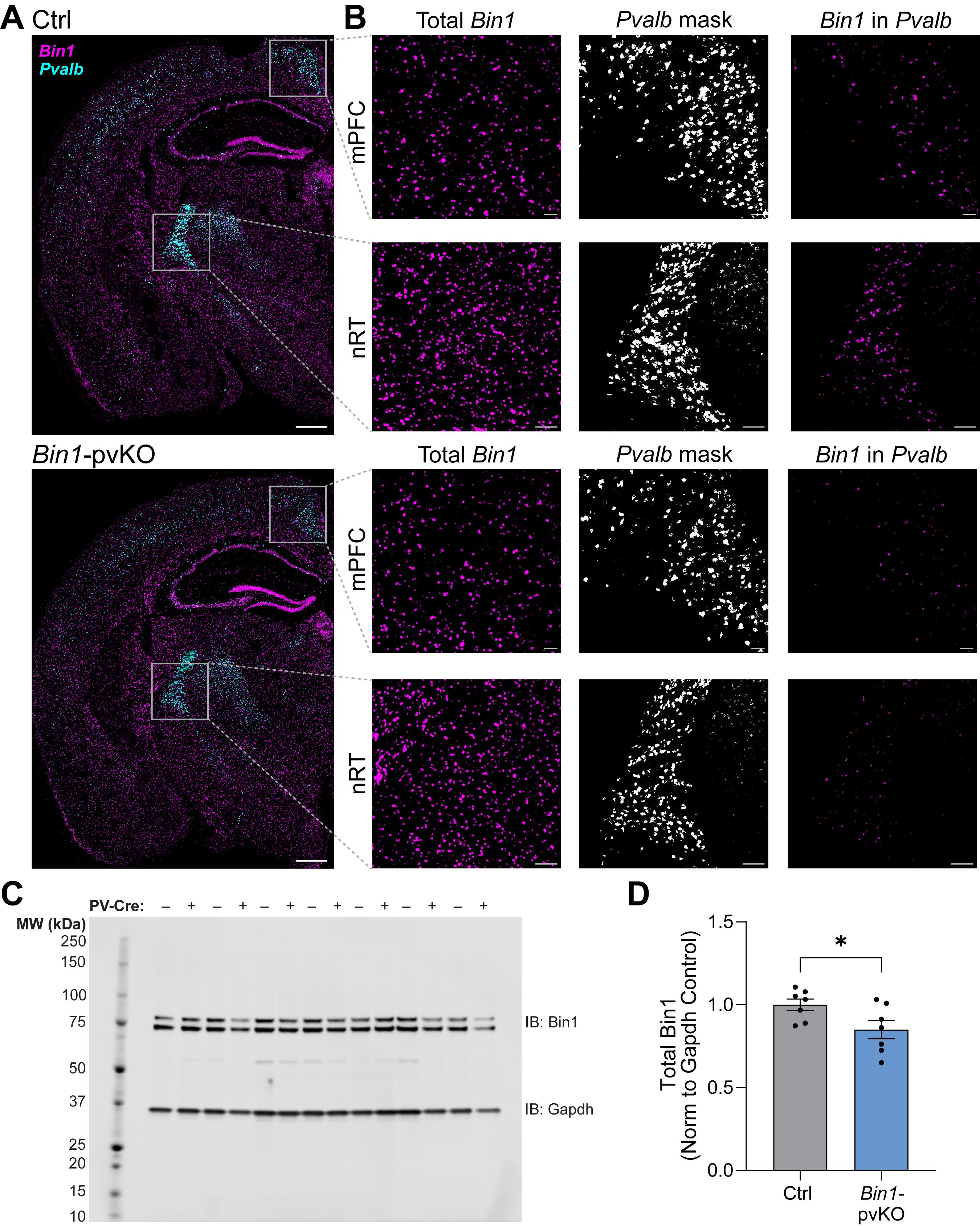

Supplement: Figure 1-2 — Selective loss of Bin1 from PV cells in Bin1-pvKO mice. A) Low power images showing smFISH of Bin1 (magenta) and Pvalb (cyan) of a control (top) and Bin1-pvKO (bottom) hemibrain, boxed insets for zoom in panel B. B) Higher power view of two PV neuron–dense regions (medial prefrontal cortex [mPFC] and reticular nucleus of the thalamus [nRT], boxed in panel A), showing total Bin1 in the region, a mask created by Pvalb + cells, and the Bin1 signal present only in the Pvalb mask, with control tissue on top and Bin1-pvKO tissue on bottom as in panel A. C) Full Western blot of Control (PV-Cre–) and Bin1-pvKO (PV-Cre+) cortical homogenate, probed for Bin1 and Gapdh. D) Quantification of total Bin1 signal normalized to Gapdh for each sample then normalized to Ctrl (unpaired t test, t (12) = 2.302, p = 0.0400, n = 7 per group, aged 5–7 months). Download Figure 1-2, TIF file. [file eneuro-13-ENEURO.0304-25.2026-s004.tif]

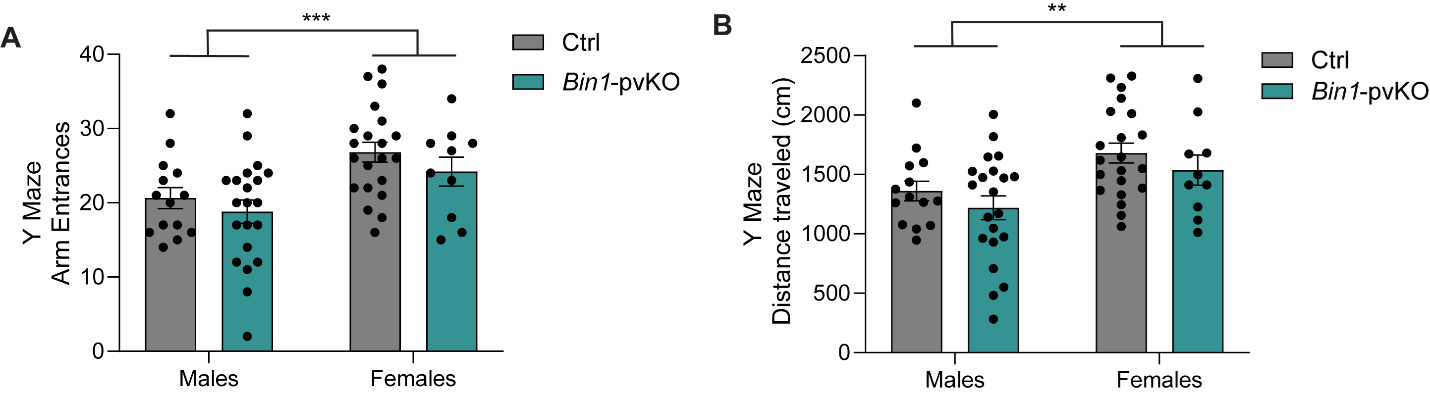

Supplement: Figure 3-1 — Exploratory behavior in the Y Maze split by sex. The differences seen in Y maze A) number of arm entrances (two-way ANOVA, interaction: F (1, 62) = 0.05641, p = 0.8131; genotype: F (1, 62) = 1.848, p = 0.1789; sex: F (1, 62) = 12.51, p = 0.0008) and B) total distance traveled (two-way ANOVA, interaction: F (1, 62) = 0.0002152, p = 0.9883; genotype: F (1, 62) = 1.923, p = 0.1705; sex: F (1, 62) = 9.577, p = 0.0030) were not driven by one sex. Download Figure 3-1, TIF file. [file eneuro-13-ENEURO.0304-25.2026-s005.tif]

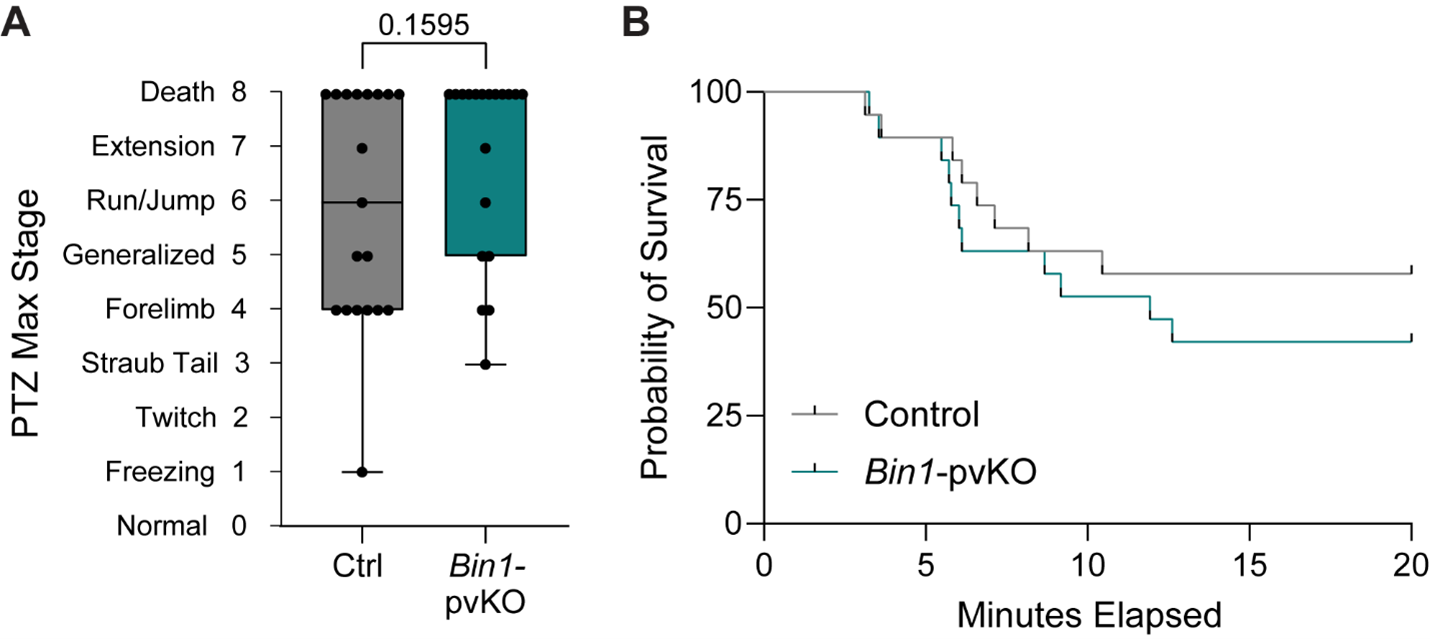

Supplement: Figure 6-1 — Both control and Bin1-pvKO mice show high seizure susceptibility at 16–20 months. A) Maximum seizure stage reached after PTZ (40 mg/kg) at 16–20 months (Mann-Whitney test, U = 136, p = 0.1595, n = 19 per group). B) Probability of survival during PTZ assay (log-rank Mantel-Cox test, Chi square = 0.8383, p = 0.3599, n = 19 per group). Download Figure 6-1, TIF file. [file eneuro-13-ENEURO.0304-25.2026-s006.tif]

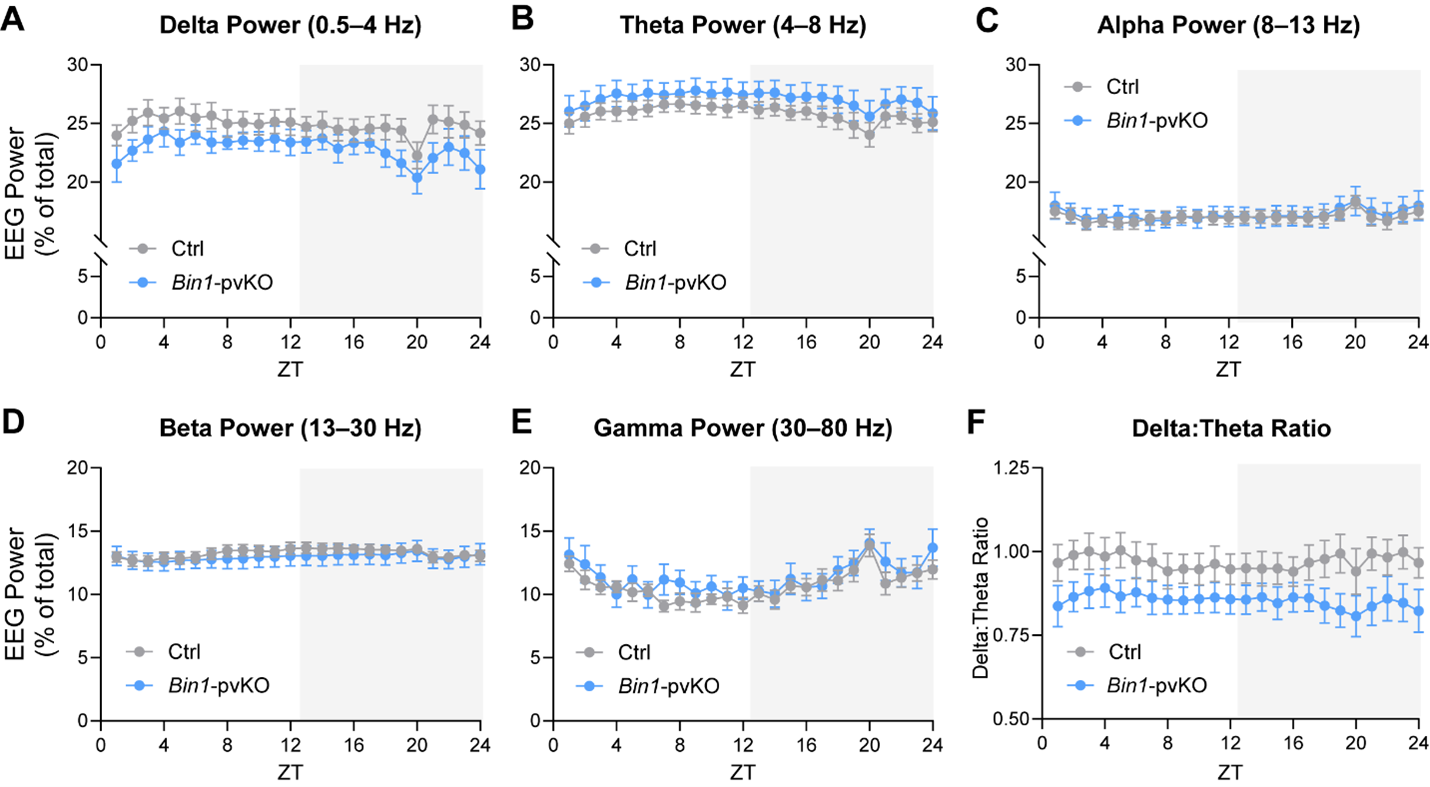

Supplement: Figure 8-1 — Individual EEG bands normalized to total power across the 24-hour cycle. EEG power as a percentage of total power for A) Delta power (two-way RM ANOVA, interaction: F (23, 276) = 0.7720, p = 0.7653, time: F (5.751, 69.01) = 4.766, p = 0.0005, genotype: F (1, 12) = 2.001, p = 0.1826), B) Theta power (two-way RM ANOVA, interaction: F (23, 276) = 0.4880, p = 0.9785, time: F (4.513, 54.16) = 8.522, p < 0.0001, genotype: F (1, 12) = 0.8628, p = 0.3713), C) Alpha power (two-way RM ANOVA, interaction: F (23, 276) = 0.4113, p = 0.9932, time: F (5.216, 62.59) = 3.640, p = 0.0053, genotype: F (1, 12) = 0.04475, p = 0.8360), D) Beta power (two-way RM ANOVA, interaction: F (23, 276) = 1.476, p = 0.0774, time: F (3.982, 47.78) = 7.490, p < 0.0001, genotype: F (1, 12) = 0.1253, p = 0.7295), and E) Gamma power (two-way RM ANOVA, interaction: F (23, 276) = 0.8836, p = 0.6210, time: F (5.688, 68.25) = 8.691, p < 0.0001, genotype: F (1, 12) = 0.3647, p = 0.5571). F) 24-hour delta:theta ratio (two-way RM ANOVA, interaction: F (23, 276) = 1.175, p = 0.2666, time: F (3.879, 46.55) = 1.648, p = 0.1797, genotype: F (1, 12) = 2.823, p = 0.1187). Download Figure 8-1, TIF file. [file eneuro-13-ENEURO.0304-25.2026-s007.tif]

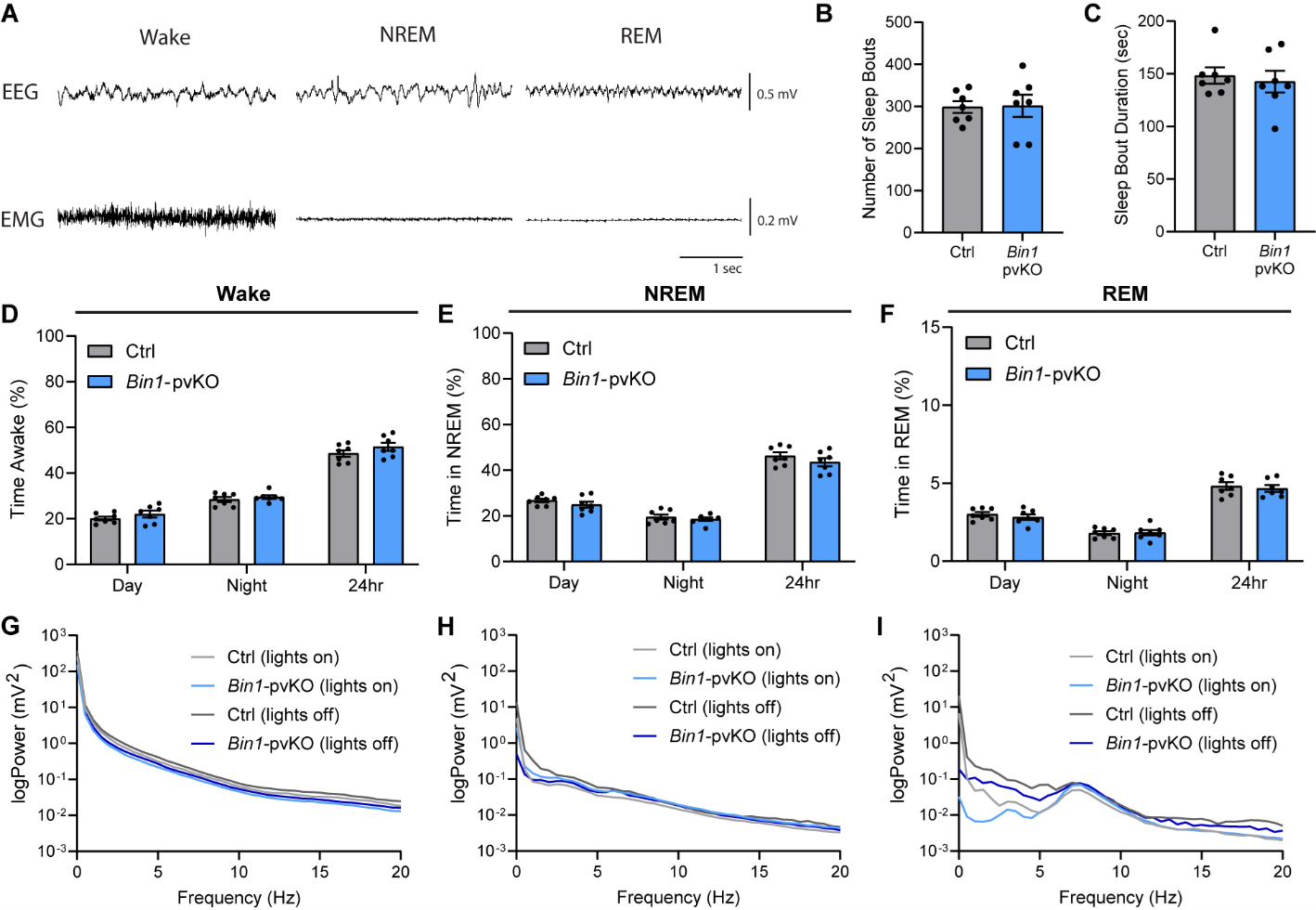

Supplement: Figure 8-2 — Bin1-pvKO mice show no overt sleep differences. A) Representative traces of EEG and EMG signal during wake, NREM, and REM states. B) Average number of sleep bouts (unpaired t test, t (12) = 0.09563, p = 0.9254). C) Average sleep bout duration (unpaired t test, t (12) = 0.4459, p = 0.6636). D) Average percent time awake during day, night, and over the 24-hour cycle (two-way RM ANOVA, time effect: F (1.678, 20.13) = 643.8, p < 0.0001, genotype effect: F (1, 12) = 1.677, p = 0.2196, interaction effect: F (2, 24) = 0.6500, p = 0.5310, n = 7 per group). E) Average percent time in NREM during day, night, and over the 24-hour cycle (two-way RM ANOVA, time effect: F (1.864, 22.37) = 590.1, p < 0.0001, genotype effect: F (1, 12) = 1.351, p = 0.2677, interaction effect: F (2, 24) = 0.6686, p = 0.5217, n = 7 per group). F) Average percent time in REM during day, night, and over the 24-hour cycle (two-way RM ANOVA, time effect: F (1.686, 20.24) = 291.8, p < 0.0001, genotype effect: F (1, 12) = 0.2187, p = 0.6484, interaction effect: F (2, 24) = 0.4309, p = 0.6548, n = 7 per group). G) Power during wake state (two-way RM ANOVA, frequency effect: F (1, 24) = 15.78, p = 0.0006; genotype effect: F (3, 24) = 0.6865, p = 0.5691, interaction effect: F (120, 960) = 0.6761, p = 0.9963, n = 7 per group). H) Power during NREM state during lights on and off (two-way RM ANOVA, frequency effect: F (1.001, 24.04) = 4.732, p = 0.0396; genotype effect: F (3, 24) = 1.283, p = 0.3028; interaction effect: F (120, 960) = 1.321, p = 0.0160; n = 7 per group). I) Power during REM state during lights on and off (two-way RM ANOVA, frequency effect: F (1.000, 24.01) = 1.684, p = 0.2068; genotype effect: F (3, 24) = 0.8456, p = 0.4825; interaction effect: F (120, 960) = 0.8313, p = 0.8998, n = 7 per group). Download Figure 8-2, TIF file. [file eneuro-13-ENEURO.0304-25.2026-s008.tif]
